# Targeting the Fra2/LCN2 axis attenuates PM_2.5_-aggravated asthma by suppressing M2 macrophage ferroptosis

**DOI:** 10.1016/j.redox.2026.104235

**Published:** 2026-05-26

**Authors:** Caihong Wang, Shutong Yang, Zhihong Zhang, Hongli Gao, Qian Niu, Jing Wu, Sanai Lv, Qi Mei, Chunyan Gao, Yukai Jing, Xiansheng Liu

**Affiliations:** a Sino-German Joint Oncological Research Laboratory, Third Hospital of Shanxi Medical University, Shanxi Bethune Hospital, Shanxi Academy of Medical Sciences, Tongji Shanxi Hospital, Taiyuan, 030032, China; b Department of Respiratory and Critical Care Medicine, Third Hospital of Shanxi Medical University, Shanxi Bethune Hospital, Shanxi Academy of Medical Science, Tongji Shanxi Hospital, Taiyuan, 030032, China; c Department of Environmental Health, School of Public Health, Shanxi Medical University, Taiyuan, 030001, China; d Department of Pharmacy, Shanxi Bethune Hospital, Shanxi Academy of Medical Sciences, Third Hospital of Shanxi Medical University, Tongji Shanxi Hospital, Taiyuan, 030032, China; e Department of Respiratory and Critical Care Medicine, Shanxi Bethune Hospital, Shanxi Academy of Medical Sciences, Third Hospital of Shanxi Medical University, Tongji Shanxi Hospital, Taiyuan, 030032, China; f Department of Emergency, First Hospital of Shanxi Medical University, Taiyuan, 030001, China; g Department of Clinical Laboratory, Shanxi Bethune Hospital, Shanxi Academy of Medical Sciences, Third Hospital of Shanxi Medical University, Tongji Shanxi Hospital, Taiyuan, 030032, China

**Keywords:** PM_2.5_, Fra2/LCN2, Macrophage, Ferroptosis, Asthma

## Abstract

Exposure to fine particulate matter (PM_2.5_) is a well-established environmental trigger of asthma exacerbations; however, the mechanistic link to macrophage ferroptosis remains elusive. This study identifies a novel pathway by which PM_2.5_ promotes asthma pathogenesis by activating the Fos-related antigen 2 (Fra2)/Lipocalin 2 (LCN2) axis, thereby inducing ferroptosis in M2 macrophages. We validated this *in vivo* using a macrophage-specific LCN2-knockdown mouse model delivered via an adeno-associated virus 9 vector. PM_2.5_-exposed M2 macrophages were analyzed *in vitro* using integrated multi-omics profiling, chromatin immunoprecipitation followed by quantitative PCR (ChIP-qPCR), transmission electron microscopy, and functional assays assessing mitochondrial integrity and ferroptosis markers. PM_2.5_ exposure disrupted macrophage polarization equilibrium, while multi-omics analyses revealed significant ferroptosis pathway enrichment and identified LCN2 as a central regulator. Mechanistically, PM_2.5_ activated the transcription factor Fra2, promoting its direct binding to the LCN2 promoter and upregulating LCN2 expression. The Fra2/LCN2 axis activation triggers ferroptosis via coordinated downregulation of FTH1 and upregulation of ACSL4 and PTGS2. This mitophagy dysfunction aggravated mitochondrial damage and intracellular iron accumulation, as evidenced by increased P62 levels and decreased LC3B levels, ultimately promoting ferroptosis in M2 macrophages. Critically, macrophage-specific knockdown of LCN2 reversed PM_2.5_-induced mitophagy inhibition and ferroptosis, thereby effectively attenuating airway inflammation and impaired lung function in asthmatic mice. Collectively, these findings reveal a previously unrecognized mechanism whereby PM_2.5_ exacerbates asthma through Fra2/LCN2-mediated mitophagy dysfunction and ferroptosis in M2 macrophages. Consequently, the Fra2/LCN2 axis may represent a potential therapeutic target for environment-associated asthma.

## Highlights

• PM_2.5_ triggers ferroptosis in M2 macrophages via the Fra2/LCN2 axis.
• The Fra2/LCN2 axis impairs mitophagy and promotes iron overload.
• Macrophage-specific LCN2 knockout reverses ferroptosis and asthma symptoms.
• The Fra2/LCN2 axis is a potential therapeutic target for PM_2.5_-aggravated asthma.

## Graphical abstract

**Figure:**
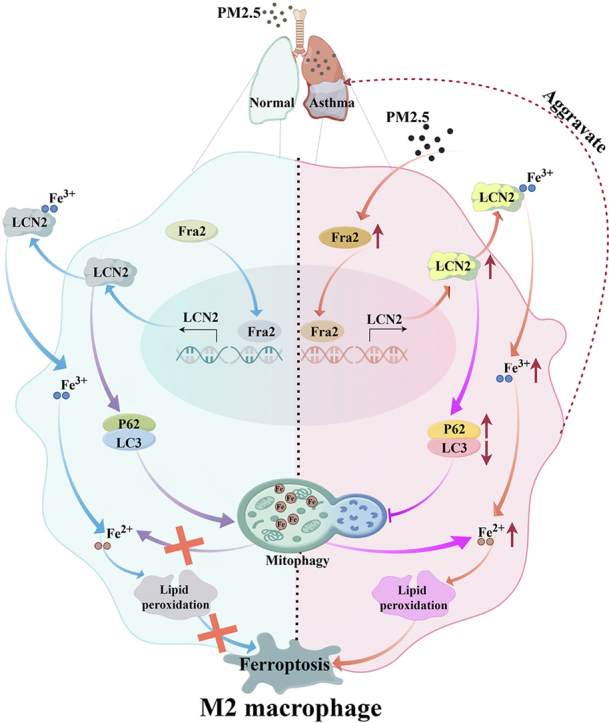

## 1 Introduction

Fine particulate matter (PM_2.5_) consists of particles with an aerodynamic diameter of 2.5 μm or less. These particles are characterized by their small size, extensive specific surface area, and high capacity for drug absorption. As a predominant pollutant in ambient air, PM_2.5_ has emerged as the leading environmental factor contributing to the global disease burden, ranking 4th among environmental risk factors in 2019 [1,2]. Notably, prolonged exposure to PM_2.5_ is linked to premature mortality from various diseases, including cardiovascular and respiratory diseases, lung cancer, and lower respiratory infections [3]. A recent study reported that each decile increase in PM_2.5_ levels was associated with a 2.1% increase in cardiovascular mortality and a 1.7% increase in respiratory mortality [4]. However, the exact pathogenesis of diseases aggravated by PM_2.5_ remains poorly understood.

Asthma is a prevalent chronic respiratory condition affecting over 300 million individuals globally and results in approximately 495,000 deaths annually, thereby imposing a significant burden on health systems and economies [5]. The disease is characterized by airway hyperresponsiveness, chronic airway inflammation, and high mucus secretion. A case-crossover study of 153 hospitals in 20 provinces of China from 2013 to 2020 demonstrated that short-term exposure to PM_2.5_ and PM_2.5-10_ was associated with increased hospital admissions for asthma [6]. Similarly, another study revealed that increased cumulative exposure to PM_2.5_ was positively correlated with increased rates of asthma emergency department visits. For instance, after eight days of cumulative exposure to PM_2.5_, a 6.3 μg/m^3^ increase in PM_2.5_ was associated with a 1.016-fold increase in rates of asthma emergency department visits (95% confidence interval [CI]: 1.007, 1.020) [7]. However, the pathogenic mechanism of PM_2.5_ aggravating asthma remain unclear.

Macrophages are key components of the innate immune system and play a pivotal role in immune surveillance and the regulation of innate and adaptive immune responses. Alveolar macrophages, the predominant immune cells in the lung responsible for processing environmental particles, constitute the first line of defense against harmful particulates [8]. Upon interaction with the local microenvironment, macrophages can differentiate into either M1 or M2 phenotypes. M1 macrophages are activated by interferon-gamma and lipopolysaccharide (LPS) and contribute to mediating pro-inflammatory responses. Conversely, M2 macrophages are induced by IL-4/IL-13 and are associated with anti-inflammatory activity, wound healing, tissue repair, and allergic inflammatory processes [9]. Previous research has demonstrated that PM_2.5_ can induce pro-inflammatory cytokine secretion in macrophages and perturb the M1/M2 balance [10,11]. Furthermore, NLRP3-mediated macrophage pyroptosis has been reported to exacerbate PM_2.5_-induced lung injury by aggravating inflammatory responses, oxidative stress, and apoptosis [12]. Meanwhile, PM_2.5_ exposure has also been demonstrated to induce macrophage senescence [13], necroptosis [14], and autophagy [15], thereby contributing to pulmonary disease progression. Collectively, these findings suggest that macrophage death modalities may play critical roles in the exacerbation of lung pathologies associated with PM_2.5_ exposures. However, whether PM_2.5_ aggravates asthma by affecting macrophage death and the specific mechanism has not been fully studied.

Ferroptosis is a regulated cell death pathway fundamentally dependent on intracellular iron accumulation and characterized by iron-catalyzed lipid peroxidation. Unlike apoptosis or autophagy, ferroptosis selectively eliminates activated cells under specific pathological conditions. Increasing evidence suggests that excessive iron accumulation in the airway epithelium of patients with asthma can induce ferroptosis, contributing to disease progression [16]. Notably, *in vivo* and *in vitro* studies have indicated that administration of the ferroptosis-specific inhibitor ferrostatin-1 (Fer-1) significantly alleviates asthma symptoms [17]. Recent studies have also indicated that PM_2.5_ can trigger ferroptosis in macrophages by modulating key ferroptosis-related proteins, leading to enhanced iron accumulation and lipid peroxidation [18]. Consistent with these findings, Kapralov et al. reported that M2 macrophages exhibit greater susceptibility to ferroptosis compared to their M0 or M1 counterparts. This difference was mainly attributed to the specific upregulation of inducible nitric oxide synthase (iNOS) in M1 macrophages, where subsequent nitric oxide (NO) production suppresses lipid peroxidation [19]. Similarly, evidence from rheumatoid arthritis studies suggests that M2 macrophages are more susceptible to ferroptosis than M1 macrophages. Ferroptosis of M2 macrophages was demonstrated to increase the M1/M2 ratio via the High-Mobility Group Box 1 (HMGB1)/Toll-like Receptor 4 (TLR4)/Signal Transducer and Activator of Transcription 3 (STAT3) signaling axis, thereby exacerbating inflammatory responses. Conversely, ferroptosis inhibitors significantly increased the M2 macrophage population and alleviated the progression of joint inflammation and structural damage [20]. However, whether PM_2.5_ exacerbates asthma by promoting M2 macrophage ferroptosis and the underlying molecular mechanisms remain unclear.

To investigate the mechanism by which PM_2.5_ induces ferroptosis in M2 macrophages and aggravates asthma, we performed integrated transcriptomic and proteomic screening. These analyses identified the Fos-related antigen 2 (Fra2)/Lipocalin 2 (LCN2) axis as the top-ranked candidate pathway. Fra-2 is a member of the activating protein-1 (AP-1) family of transcription factors (TFs) and plays a crucial role in regulating cell growth and differentiation. Previous studies have linked Fra2 to multiple immune and respiratory systems [21], and increased Fra2 expression and activation have been reported in several pulmonary diseases [22,23]. LCN2, also known as neutrophil gelatinase-associated lipocalin (NGAL) or siderocalin, belongs to the lipocalin superfamily. As a circulating protein, LCN2 participates in regulating various biological processes in eukaryotic cells. Under stress conditions such as infection and tissue injury, LCN2 contributes to inflammatory and other cellular stress responses by modulating iron metabolism [24]. In the lung, LCN2 is associated with acute inflammation and oxidative stress and has been demonstrated to promote neutrophil recruitment and activate pro-inflammatory cytokine signaling pathways [25].

Based on these screening results, we next investigated the functional role of the Fra2/LCN2 axis using both *in vivo* (a PM_2.5_-aggravated asthmatic mouse model) and *in vitro* (PM_2.5_-exposed macrophage culture) models. Our findings demonstrate that PM_2.5_ activates the Fra2/LCN2 axis, leading to impaired mitophagy, intracellular iron accumulation, and ferroptosis in M2 macrophages. The resultant disruption of M1/M2 macrophage homeostasis contributes to asthma exacerbation. Our findings establish a novel mechanistic foundation for PM_2.5_-induced asthma aggravation and identify potential targets for therapeutic intervention against PM_2.5_-related asthma exacerbations.

## 2 Materials and methods

### 2.1 PM_2.5_ collection and preparation

Between November 2022 and January 2023, PM_2.5_ samples were gathered using an automated high-volume air sampler (TH-1000CII, Tianhong, Wuhan, China) with a PM_2.5_ impactor, positioned on the rooftop of the School of Public Health at Shanxi Medical University in Taiyuan, Shanxi Province, China. The PM_2.5_ filter samples were sliced into small squares measuring 1 cm × 1 cm. These were then subjected to three 20-min cycles of sonication using an ultrasonic cleaner (Kunshan Ultrasonic Instrument Co., Ltd., Jiangsu, China). The preparation of the PM_2.5_ samples involved filtering the suspensions through sterile gauze, followed by lyophilization with a vacuum freeze dryer (Thermon Anderson, USA). The physicochemical characteristics, including size distribution and composition, of the PM_2.5_ samples were consistent with our previous findings [26].

### 2.2 Establishment of an asthma mouse model exacerbated by PM_2.5_

Male C57BL/6J mice (6–8 weeks old) were obtained from SPF Biotechnology Co., Ltd. (Beijing, China) and housed under standard conditions with a 12-h light/dark cycle. After a one-week acclimation period, all experiments were conducted in accordance with the protocols approved by the Laboratory Animal Ethics Committee of the Shanxi Cancer Hospital (Approval No. 2022026).

Following acclimatization, 60 mice were randomly assigned into six groups as follows: normal control group (NC), ovalbumin group (OVA), OVA + PM_2.5_ group (PM_2.5_), OVA + PM_2.5_ + Ferrostatin-1 group (PM_2.5_ + Fer-1) (n = 10 per group), OVA + PM_2.5_ + AAV9-F4/80-Control group (PM_2.5_ + AAV-Ctrl), OVA + PM_2.5_ + AAV9-F4/80-RNAi-LCN2 group (PM_2.5_ + AAV-RNAi-LCN2) (n = 11 per group).

For sensitization, mice in the OVA groups received intraperitoneal injections of 0.2 mL OVA suspension on days 0, 7, and 14, respectively. The suspension contained 25 μg of OVA (Sigma, USA) adsorbed onto 1 mg of aluminum hydroxide (Al(OH)_3_). Starting on day 21, an airway challenge was administered every other day via nebulization of 2% OVA, with each session lasting for 30 min, for a total of 10 exposures. Mice in the NC group were administered PBS via intraperitoneal injection and nebulized challenge as controls. Mice in the PM_2.5_ + Fer-1 group received an intraperitoneal injection of the ferroptosis inhibitor, ferrostatin-1 (1 mg/mL) 1 h before nebulization.

Beginning on day 20, all mice in the PM_2.5_ exposure groups were anesthetized with isoflurane and subsequently received intranasal instillation of 20 μL PM_2_._5_ suspension at a concentration of 3.6 mg/kg·BW every other day for a total of 10 administrations (refer to our previously published article [26]). Mice in the NC and OVA groups were intranasally instilled with 20 μL of blank filter extract, which served as the solvent control for the PM_2.5_ suspension.

Macrophage-specific LCN2 knockdown mice were established via intratracheal instillation of an adeno-associated virus serotype 9 (AAV9) vector carrying a macrophage-specific promoter (F4/80) and encoding LCN2 siRNA, with AAV9-F4/80-Ctrl administered intratracheally as a control. The viral dose was 1.0 × 10^12^ genomic copies per mouse, and all viruses were purchased from GenePharma (Shanghai, China).

### 2.3 Cell culture

The RAW264.7 (RRID: CVCL_0493) macrophage cell line was obtained from Shanghai Zhong Qiao Xin Zhou Biotechnology Co., Ltd. (Cat# ZQ0098, Shanghai, China) and cultured in high-glucose DMEM supplemented with 10% FBS under standard conditions (37 °C, 5% CO_2_). M2 polarization was induced by treating the cells with 20 ng/mL interleukin-4 (IL-4) for 12 h. The optimal exposure concentration of PM_2.5_ was determined to be 50 μg/mL based on the CCK-8 assay (Fig. S9), and all subsequent experiments were conducted 12 h after PM_2.5_ exposure. Cells were pre-treated with 10 μM Ferrostatin-1 (Fer-1) for 3 h before exposure to IL-4 and PM_2.5_ (the impact of Fer-1 on viability is shown in Figs. S17 and S18). Additionally, transfection with Fra2 and LCN2-targeting siRNA (Sangon Biotech, Shanghai, China) was performed using the Lipofectamine™ 3000 (Invitrogen, CA, USA) manufacturer's protocol.

THP-1 cells (RRID: CVCL_0006) were purchased from Shanghai Zhong Qiao Xin Zhou Biotechnology Co. Ltd. (Cat# ZQ0086, Shanghai, China) and cultured in RPMI-1640 medium containing 10% FBS under standard conditions (37 °C, 5% CO_2_). To differentiate THP-1 cells into macrophage-like cells, they were seeded in appropriate culture vessels and treated with 100 nM phorbol 12-myristate 13-acetate (PMA) for 72 h, followed by stimulation with 20 ng/mL IL-4 for 48 h to promote M2 polarization. PM_2.5_ and Fer-1 treatments were applied using the same protocols as those used for RAW264.7 cells.

All cell lines used in this study were confirmed to be free of mycoplasma contamination and were routinely tested using PCR.

### 2.4 Transcriptomic and proteomic sequencing and data analysis

RAW264.7 cells stimulated with PM_2.5_ for 12 h were collected for transcriptomic and proteomic sequencing (HuaYing Bio, Shanghai, China). The raw transcriptomic read count data were normalized using the median-of-ratios method implemented in DESeq2, and gene expression levels were presented as Transcripts Per Million (TPM). Sample expression distributions and batch effects were evaluated using box plots, Pearson correlation heatmaps, and principal component analysis (PCA) (Fig. S33). Differential expression analysis was performed based on a negative binomial distribution model, followed by multiple hypothesis testing correction using the Benjamini-Hochberg method. Differentially expressed genes were identified with |log2(Fold Change)| > 1 and padj <0.05. For proteomic data, protein identification was quality-controlled based on the Protein FDR Confidence: Combined column, and only proteins with FDR <0.01 (high confidence) were retained for subsequent analyses. Differentially expressed proteins were defined as those with |log2(Fold Change)| > 1 and *P* < 0.05. Both transcriptomic and proteomic data were corrected for batch effects using the ComBat method prior to differential analysis. Venn diagrams were generated using Venny 2.1 to identify consistently differentially expressed molecules at both transcriptional and translational levels. A series of bioinformatic analyses, including functional annotation, Gene Ontology (GO), Kyoto Encyclopedia of Genes and Genomes (KEGG) enrichment, and Protein-Protein Interaction (PPI) network construction, were conducted to elucidate the biological functions and pathways of the identified molecules. Furthermore, the publicly available dataset GSE74986 was analyzed to compare the expression of LCN2 and Fra2 in bronchoalveolar lavage fluid (BALF) cells from patients with asthma and controls.

### 2.5 Pathological analysis

After fixation with 4% paraformaldehyde, the lung tissues underwent standard processing for dehydration, paraffin embedding, and microtome sectioning at 3–5 μm. Tissue sections were then subjected to H&E staining to analyze inflammatory infiltration and PAS staining to detect goblet cell hyperplasia and mucus secretion. Quantitative analysis was performed by calculating the inflammatory cell infiltration area on H&E-stained sections and the percentage of PAS-positive area on PAS-stained sections using ImageJ software.

### 2.6 Determination of pulmonary function

One day prior to euthanasia, the mice were acclimatized for 30 min in the chamber of a whole-body plethysmograph (WBP-4MR; Tow-int, Shanghai, China). Unrestrained mice were subsequently monitored for 20 min to record the following parameters: tidal volume (Vt), respiratory frequency (f), inspiratory time (Ti), expiratory time (Te), minute ventilation (MV), mid-expiratory flow (EF50), and enhanced pause (Penh).

### 2.7 Immunofluorescence

The tissue sections were deparaffinized and rehydrated using a graded ethanol series. After blocking for 1 h at room temperature, the sections were probed with primary antibodies overnight at 4 °C. Subsequently, the sections were incubated with fluorescence-conjugated secondary antibodies for 1 h at room temperature, protected from light. Nuclei were counterstained with DAPI for 10 min at room temperature under light-protected conditions. Finally, the sections were mounted with an anti-fade mounting medium and imaged using a fluorescence microscope. Quantitative and colocalization analyses were performed using ImageJ software. The mean fluorescence intensity was measured, and colocalization was assessed using Manders' overlap coefficients (M1/M2), calculated using the JACoP plugin.

### 2.8 Transmission electron microscope

For mitochondrial ultrastructural analysis, RAW264.7 cells were fixed in 2.5% glutaraldehyde (4 °C, 12 h), rinsed with PBS, and post-fixed with 1% osmium tetroxide (4 °C, 2 h). The samples were then dehydrated using a graded ethanol series, transitioned with acetone, and embedded in Epon resin for polymerization (60 °C, 48 h). Ultrathin sections (50–70 nm) prepared with an ultramicrotome were double-stained with uranyl acetate and lead citrate prior to observation under a transmission electron microscope. Quantitative analysis of mitochondrial morphology was performed using the ImageJ software. At least 30 randomly selected mitochondria were analyzed for each group. The mitochondrial volume was estimated by measuring the cross-sectional area of each mitochondrion. Mitochondrial electron density was assessed by measuring the mean gray value within the mitochondrial region.

### 2.9 Flow cytometry analysis

Following harvest, THP-1 and RAW264.7 cells were labeled with Fixable Viability Stain 620 (564996, BD Biosciences, USA) to discriminate live from dead cells, followed by Fc receptor blocking. After fixation/permeabilization using a commercial kit, intracellular staining was performed with Allophycocyanin (APC) anti-human CD206 (321109, BioLegend, USA) or Alexa Fluor™ 647 Anti-Mouse CD206 (568809, BD Biosciences, USA) antibody for 30 min at 4 °C in the dark. For mouse BALF cells, after staining with Fixable Viability Stain 620 and blocking Fc receptors, surface staining was performed using Fluorescein Isothiocyanate (FITC)-anti-F4/80 (CFE8001, Cohesion Biosciences, Suzhou, China) and PE-anti-CD86 (105007, BioLegend, USA). Following fixation and permeabilization, intracellular staining was conducted with Alexa Fluor™ 647-conjugated anti-mouse CD206 (568809, BD Biosciences, USA), together with primary antibodies against LCN2 (31452-1-AP, Proteintech, Wuhan, China), ACSL4 (FNab00108, FineTest, Wuhan, China), and LC3B (FNab04720, FineTest, Wuhan, China). Cells were subsequently incubated with Alexa Fluor™ Plus 405-conjugated goat anti-rabbit Immunoglobulin G (IgG) secondary antibody (A48254, Invitrogen, USA). Fluorescence-minus-one controls were used to establish gating boundaries between background fluorescence and positive populations. Analysis was performed using a Beckman CytoFLEX flow cytometer, and data were processed with FlowJo software.

### 2.10 Real-time quantitative PCR

Following total RNA extraction from cells using TRIzol® reagent (Takara, Japan), cDNA was synthesized using a reverse transcription kit (PrimeScript™ RT with gDNA Eraser). Quantitative PCR was performed using the TB Green® Premix Ex Taq™ kit (Takara, Japan) and gene-specific primers (sequences in Supplementary Tables S2–S3, Sangon Biotech, Shanghai, China). Gene expression was quantified via the 2^−ΔΔCt^ method, normalizing to β-actin.

### 2.11 Iron analysis

The ferrous ion content detection kit (G1217W, Grace Biotech; China) was used to measure ferrous ion levels. In accordance with the manufacturer's instructions, cultured cells were first collected, and the extraction solution was added for ultrasonic disruption. After 10 min of centrifugation at 12,000 rpm and 4 °C, the supernatant was collected and maintained on ice for subsequent analysis. For mouse serum and BALF, the supernatant can be used directly or collected after centrifugation and placed on ice for detection. Standard and blank tubes were prepared according to the kit protocol and transferred to 1 mL glass cuvettes, along with the experimental samples, for analysis. Absorbance was measured at 562 nm for all tubes. Additionally, intracellular Fe^2+^ was measured using the FerroOrange probe (C8004, APExBIO, USA). FerroOrange was diluted to a final concentration of 1 μM working solution according to the manufacturer's instructions. Collected cells were incubated with the FerroOrange working solution for 30 min at 37 °C in the dark, then immediately analyzed by flow cytometry.

### 2.12 BODIPY™ 581/591 C11 fluorescent probe staining

The cells were collected and washed once with 1× PBS. According to the instructions of the BODIPY™ 581/591 C11 kit (GC40165, Glpbio, USA), 100 μL of BODIPY™ 581/591 C11 probe at a final concentration of 2 μM was added to each sample, followed by incubation at 37 °C for 30 min in the dark. The cells were then washed twice with 1× PBS and analyzed using the FITC channel of a flow cytometer.

### 2.13 Detection of malondialdehyde (MDA) and glutathione (GSH)

To assess lipid peroxidation, the concentrations of malondialdehyde (MDA) and glutathione (GSH) in cell lysates, mouse serum, and BALF were quantified using commercial assay kits (A003-2 and A006-2, Nanjing Jiancheng Bioengineering Institute, China) according to the manufacturer's protocols.

### 2.14 Western blot

RAW264.7 and THP-1 cells were lysed using RIPA buffer. Total protein concentration was quantified using a BCA protein assay kit. The protein samples were separated by 6%–10% sodium dodecyl sulfate–polyacrylamide gel electrophoresis (SDS-PAGE) and subsequently transferred onto polyvinylidene fluoride (PVDF) membranes. The membranes were blocked with 5% skim milk at room temperature for 1 h, followed by incubation with primary antibodies at 4 °C overnight. The primary antibodies used were as follows: CD206 (FNab01442, FineTest, Wuhan, China), Arg1 (16001-1-AP, Proteintech, Wuhan, China), ACSL4 (FNab00108, FineTest, Wuhan, China), PTGS2 (sc-376861, Santa Cruz, USA), FTH1 (sc-376594, Santa Cruz, USA), Fra2 (R389230, Zenbio, China), LCN2 (31452-1-AP, Proteintech, Wuhan, China), P62 (84826-1-RR, Proteintech, Wuhan, China), LC3B (FNab04720, FineTest, Wuhan, China), β-actin (66009-1, Proteintech, Wuhan, China). After probing with horseradish peroxidase (HRP)-conjugated secondary antibodies (1 h, room temperature), immunoreactive bands were detected using enhanced chemiluminescence (ECL) (SEVEN, China). Quantitative analysis was performed using the ImageJ software. Band intensities were measured and normalized to β-actin, which was used as an internal control.

### 2.15 Chromatin immunoprecipitation followed by quantitative PCR (ChIP-qPCR)

The ChIP assay was performed using a commercial kit (Cat. No. RK20258, Abclonal, China) according to the manufacturer's instructions. Briefly, samples were cross-linked with 1% formaldehyde under appropriate conditions. Cells were then lysed using Cell Swelling Buffer and sonicated in ChIP Sonication Buffer to fragment chromatin. To evaluate chromatin fragmentation efficiency, a 50 μL aliquot of the sonicated lysate was reserved for DNA extraction after reverse cross-linking. For each immunoprecipitation, 5% of the sonicated sample was retained as the Input control. The residual lysate was aliquoted and probed with the Fra2 antibody, a positive control antibody against Histone H3 (Cat. No. RM20711), and a negative control normal IgG antibody (Cat. No. RM20712). Immunoprecipitated complexes were collected, washed, and eluted using ChIP Elution Buffer. Reverse cross-linking and DNA purification were subsequently performed according to the manufacturer's protocol. Quantitative real-time PCR (qPCR) was carried out to analyze the precipitated DNA fragments. The qPCR reactions included Input, positive control (H3), negative control (IgG), and LCN2. Results were normalized to the Input sample and analyzed using the 2^−ΔΔCt^ method.

### 2.16 Measurement of intracellular reactive oxygen species (ROS)

Intracellular ROS levels were measured using a ROS assay kit with the fluorescent probe 2′,7′-Dichlorodihydrofluorescein Diacetate (DCFH-DA) (Leagene Biotechnology, Beijing, China). Following cellular uptake, DCFH-DA is hydrolyzed by intracellular esterases to generate DCFH, which is subsequently oxidized by ROS to form the highly fluorescent compound 2′,7′-Dichlorofluorescein (DCF). RAW264.7 cells were plated in 12-well plates. Before assay, the supernatant was removed, and cells were incubated with 1 mL of 10 μM DCFH-DA working solution for 20 min incubation at 37 °C in the dark. Fluorescence signal intensity was measured using a fluorescence microscope.

### 2.17 Mitochondrial membrane potential assay kit with JC-1

JC-1 staining working solution and carbonyl cyanide m-chlorophenyl hydrazone (CCCP) solution for the positive control were prepared as directed by the manufacturer (Leagene Biotechnology, Beijing, China). Following a 20-min incubation with JC-1 working solution at 37 °C, RAW264.7 cells were washed twice with assay buffer prior to fluorescence imaging. The mitochondrial membrane potential (ΔΨm) was determined using the red-to-green fluorescence intensity ratio.

### 2.18 Colocalization of mitochondria and LC3B

Cells were treated with 200 nM MitoTracker™ Red CM-H2XRos (M7513, Invitrogen) at 37 °C for 30 min, fixed with 4% paraformaldehyde for 15 min, and permeabilized with 0.2% Triton X-100 for 10 min. After a 1-h room temperature block, they were incubated overnight at 4 °C with an LC3B primary antibody (FNab04720, FineTest, Wuhan, China), followed by a 1-h dark incubation with an Alexa Fluor™ 488-conjugated goat anti-rabbit IgG secondary antibody (R37116, Invitrogen, USA). Nuclei were stained with DAPI for 5 min, and the samples were mounted and examined using a confocal microscope.

### 2.19 Statistical analysis

Statistical analysis was performed using the Statistical Package for the Social Sciences software (version 22.0), while graphical representations were generated with GraphPad Prism software (version 9.0). All data are presented as mean ± standard deviation (SD). Data normality was assessed using the Shapiro–Wilk test. Parametric statistical analyses were applied only to datasets demonstrating a normal distribution (*P* > 0.05). For non-normally distributed data, non-parametric tests were applied. Specifically, comparisons between two groups were performed using the Mann–Whitney *U* test, whereas comparisons among multiple groups were performed using the Kruskal–Wallis test. For normally distributed datasets, comparisons between two groups were performed using an unpaired two-tailed Student's *t*-test. For comparisons among multiple independent groups, one-way analysis of variance was used, followed by Tukey's post hoc test. Pearson correlation analysis was employed to assess the associations between PM_2.5_ concentration and lung function parameters, serum total IgE levels, and the proportions of macrophages, eosinophils, and neutrophils in BALF from patients with asthma. Statistical significance was defined as *ns* (non-significant), *P < 0.05, and **P < 0.01.

## 3 Results

### 3.1 PM_2.5_-induced M1/M2 macrophage imbalance contributes to asthma exacerbation

**Fig. 1:**
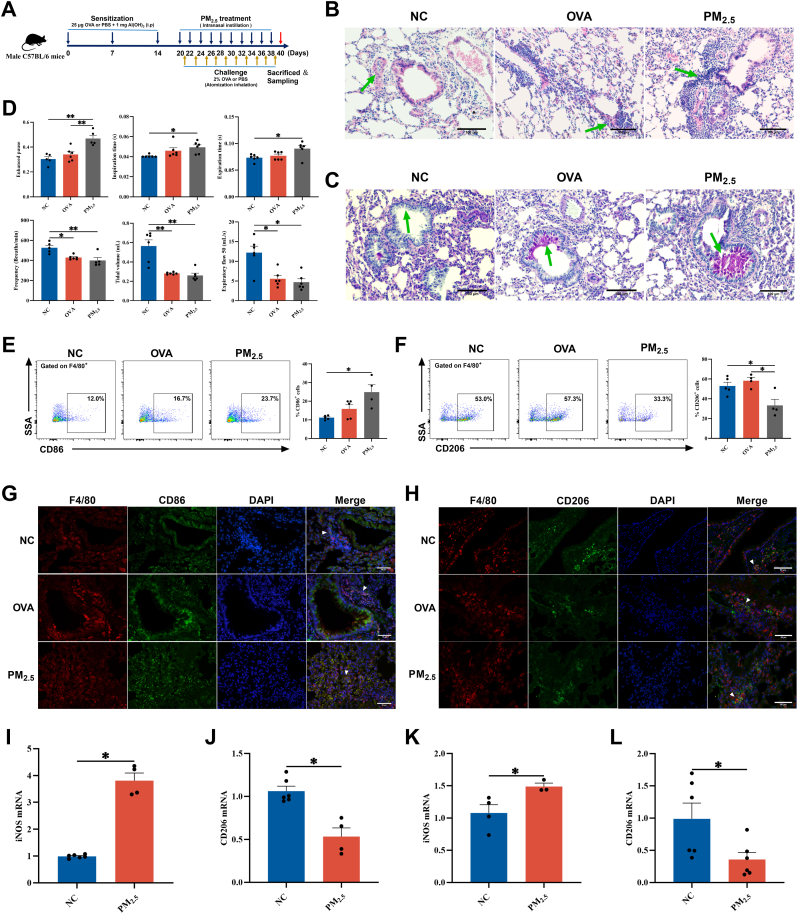
**PM_2.5_ induces M1/M2 macrophage imbalance in asthma. (A)** Schematic diagram for the establishment of a mouse model of PM_2.5_ exacerbating asthma. **(B)** Representative photomicrographs of H&E-stained lung tissue sections. Scale bar: 100 μm. Green arrows indicate infiltrating inflammatory cells. **(C)** Representative images of PAS-stained lung tissue sections. Scale bar: 100 μm. Green arrows denote secreted mucus and proliferated goblet cells. **(D)** Lung function of mice (n = 6). **(E)** Flow cytometry was used to analyze the proportion of CD86^+^ cells in mouse BALF (n = 6). **(F)** Flow cytometry quantified the proportion of CD206^+^ cells in mouse BALF (n = 6). **(G)** Immunofluorescence staining for CD86 in mouse lung tissue. Scale bar: 50 μm. **(H)** Immunofluorescence staining for CD206 in mouse lung tissue. Scale bar: 50 μm. **(I)** The mRNA level of iNOS was detected by RT-qPCR in PM_2.5_-stimulated RAW264.7 cells (n = 6). **(J)** CD206 mRNA levels were measured by RT-qPCR in PM_2.5_-stimulated RAW264.7 cells (n = 6). **(K)** The mRNA levels of iNOS detected by RT-qPCR in PM_2.5_-stimulated THP-1 cells (n = 6). **(L)** CD206 mRNA levels were measured by RT-qPCR in PM_2.5_-stimulated THP-1 cells (n = 6). Values are expressed as the means ± SD. * indicates *P* < 0.05 and ** indicates *P* < 0.01.

Initially, an Ovalbumin (OVA)-sensitized and aerosol-challenged murine asthma model was established, followed by intranasal instillation of PM_2.5_ suspensions to induce PM_2.5_-aggravated asthma (Fig. 1A). As illustrated in Figs. S1, S2A, and S3 mice exposed to PM_2.5_ exhibited significantly lower body weight and increased organ coefficients for the heart, liver, and lung compared to the control group. Since IgE is a key mediator of allergic responses, elevated serum OVA-specific IgE (OVA-sIgE) levels were used to confirm successful OVA-induced asthma in the model. Enzyme-Linked Immunosorbent Assay (ELISA) analysis indicated elevated OVA-sIgE levels in the OVA and PM_2.5_ groups relative to the NC group (*P* < 0.01) (Fig. S4A). Histopathological analysis via hematoxylin and eosin (H&E) and Periodic Acid-Schiff (PAS) staining demonstrated that, compared to the control group, the OVA group indicated thickened bronchial walls, pronounced perivascular and submucosal inflammatory cell infiltration, prominent goblet cell hyperplasia, and excessive mucus secretion. These pathological features were further aggravated in the PM_2.5_ group, including severe inflammatory cell infiltration, exacerbated goblet cell hyperplasia, and significantly enhanced mucus secretion in the airway lumen (Fig. 1B–C, S7). Pulmonary function was subsequently evaluated using whole-body plethysmography. Furthermore, PM_2.5_ exposures exacerbated lung function impairment in asthmatic mice. In comparison with the NC group, the PM_2.5_ group exhibited significantly prolonged inspiratory time (Ti) and expiratory times (Te), increased enhanced pause values, and decreased respiratory frequency (F), tidal volume, and expiratory flow at 50% tidal volume values (Fig. 1D). Airway resistance (RL) further confirmed significant lung dysfunction. As illustrated in Fig. S5, RL was significantly elevated in the OVA group compared with the NC group, while PM_2.5_ exposures further exacerbated RL in asthmatic mice. To evaluate the effect of PM_2.5_ exposures on airway inflammation, we analyzed total leukocyte counts as well as the percentages of macrophages and lymphocytes in BALF from each group of mice. These findings confirmed successful asthma induction and demonstrated that PM_2.5_ exposure worsens asthma severity. Correlation analyses were additionally performed to evaluate the relationship between PM_2.5_ exposure concentration and pulmonary function in patients with asthma. As illustrated in Fig. S8, PM_2.5_ exposure was significantly negatively correlated with Forced Expiratory Volume (FEV)1% predicted (FEV1%) and FEV1/Forced Vital Capacity (FVC)%, while displaying positive correlations with serum total IgE levels and FeNO. Furthermore, PM_2.5_ exposure was negatively associated with the proportion of macrophages in BALF, although no significant correlations were observed with eosinophils or neutrophil proportions. These findings suggest that PM_2.5_ exposure exacerbates symptoms in patients with asthma.

PM_2.5_ exposures also significantly increased total leukocyte counts and the proportions of lymphocytes in murine BALF while markedly reducing the macrophage population (Fig. S6). Moreover, flow cytometry analysis of BALF displayed that PM_2.5_ exposures increased the proportion of CD86^+^ (M1) macrophages while reducing CD206^+^ (M2) macrophages (Fig. 1E–F). Immunofluorescence staining of lung tissue further confirmed enhanced colocalization of F4/80 with CD86 and decreased colocalization with CD206 upon PM_2.5_ exposures (Fig. 1G–H, S32A).

*In vitro*, a Cell Counting Kit (CCK)-8 assay was performed to assess cell viability following a 12 h treatment with different doses of PM_2.5_. Cell viability progressively decreased as PM_2.5_ concentration increased and reached approximately 70% at 50 μg/mL (Fig. S9). Consequently, a concentration of 50 μg/mL was selected as the working concentration for the following experiments. At this concentration, PM_2.5_-stimulated RAW264.7 cells exhibited upregulated iNOS (an M1 marker) and downregulated CD206 (an M2 marker) expression (Fig. 1I–J). Similar findings were observed in PM_2.5_-stimulated THP-1 cells (Fig. 1K–L). These results indicate that PM_2.5_ exposure promotes M1 macrophage polarization while suppressing M2 polarization, thereby disrupting M1/M2 balance and potentially contributing to aggravated airway inflammation and asthma progression.

### 3.2 PM_2.5_ drives M2 macrophage ferroptosis

**Fig. 2:**
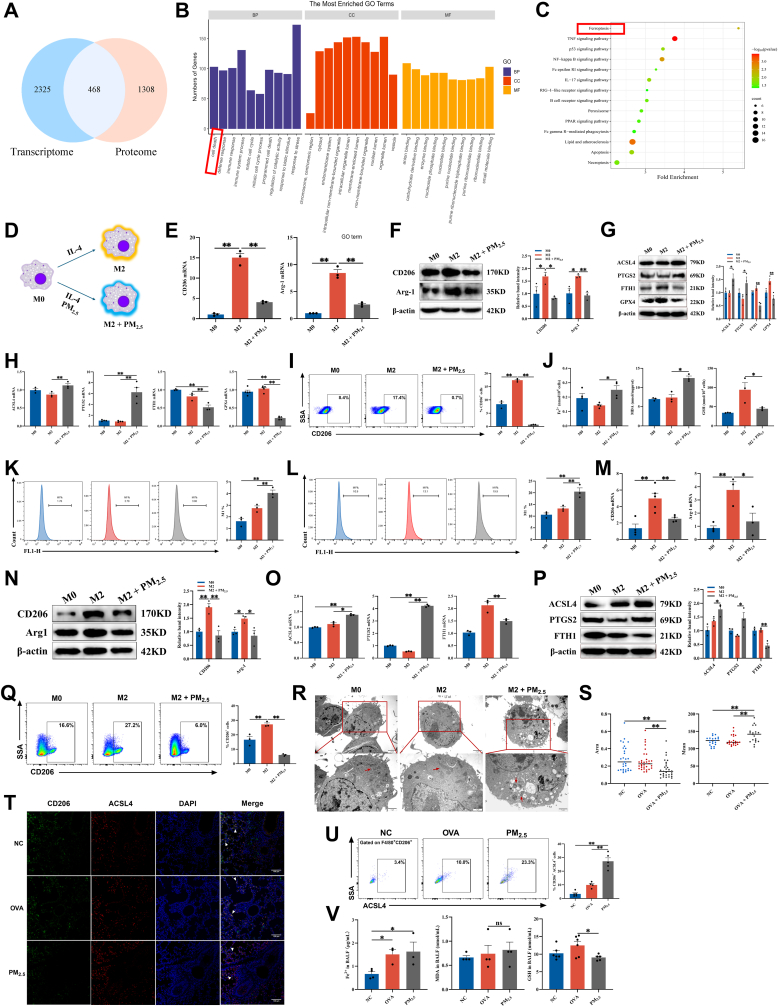
**PM_2.5_ drives M2 macrophage ferroptosis. (A)** Venn diagram of the transcriptomic and proteomic profiles of RAW264.7 cells stimulated with PM_2.5_ for 12 h. **(B)** GO enrichment analysis of the 468 DEGs. **(C)** KEGG pathway enrichment analysis of the 468 DEGs. **(D)** Schematic of M2 macrophage stimulated by PM_2.5_*in vitro*. **(E)** RT-qPCR detected the mRNA levels of CD206 and Arg1 in PM_2.5_-stimulated M2 macrophages derived from RAW264.7 cells (n = 3). **(F)** The protein levels of CD206 and Arg1 were detected by Western blot in PM_2.5_-stimulated M2 macrophages derived from RAW264.7 cells (n = 3). **(G)** Western blot was used to measure the protein levels of ACSL4, PTGS2, GPX4, and FTH1 in PM_2.5_-stimulated M2 macrophages derived from RAW264.7 cells (n = 3). **(H)** RT-qPCR detected the mRNA levels of ACSL4, PTGS2, GPX4, and FTH1 in PM_2.5_-stimulated M2 macrophages derived from RAW264.7 cells (n = 5). **(I)** Flow cytometry analysis of the proportion of CD206^+^ cells in PM_2.5_-stimulated M2 macrophages derived from RAW264.7 cells (n = 3). **(J)** Measurement of Fe^2+^, MDA, and GSH levels in PM_2.5_-stimulated RAW264.7 cell-derived M2 macrophages (n = 4). **(K)** Flow cytometry analysis of cells stained with FerroOrange (n = 3). **(L)** Flow cytometry analysis of cells stained with BODIPY™ 581/591 C11 (n = 3). **(M)** RT-qPCR detected the mRNA levels of CD206 and Arg1 in PM_2.5_-stimulated THP-1 cell-derived M2 macrophages (n = 5). **(N)** The protein levels of CD206 and Arg1 were detected by Western blot in PM_2.5_-stimulated THP-1 cell-derived M2 macrophages (n = 3). **(O)** RT-qPCR detected the mRNA levels of ACSL4, PTGS2, and FTH1 in PM_2.5_-stimulated THP-1 cell-derived M2 macrophages (n = 3). **(P)** The protein levels of ACSL4, PTGS2, and FTH1 were detected by Western blot in PM_2.5_-stimulated THP-1 cell-derived M2 macrophages (n = 3). **(Q)** Flow cytometry quantified the proportion of CD206^+^ cells in PM_2.5_-stimulated M2 macrophages derived from THP-1 cells (n = 3). **(R)** Representative transmission electron microscope (TEM) images of PM_2.5_-stimulated M2 macrophages derived from RAW264.7 cells. Scale bar: 2 μm. Red arrows indicate mitochondria. **(S)** Quantitative analysis of mitochondrial volume and density (n = 20–30). **(T)** Immunofluorescence staining for CD206 and ACSL4 in mouse lung tissue. Scale bar: 100 μm. **(U)** Flow cytometry analysis was used to analyze the proportion of CD206^+^ACSL4^+^ cells in mouse BALF (n = 5). **(V)** Measurement of Fe^2+^, MDA, and GSH levels in mouse BALF (n = 6). Values are expressed as the means ± SD. * indicates *P* < 0.05, ** indicates *P* < 0.01 and ns for no significance.

We performed integrated transcriptomic and proteomic profiling of PM_2.5_-stimulated RAW264.7 cells and identified 468 co-differentially expressed genes (DEGs) (∣log_2_FC∣> 1, *P* < 0.05) (Fig. 2A). Volcano plots and cluster heatmaps of the DEGs from the transcriptomic and proteomic analyses are illustrated in Supplementary Fig. S10–11. Gene ontology (GO) enrichment analysis revealed significant enrichment of biological processes related to cell death. Consistently, Kyoto encyclopedia of genes and genomes (KEGG) pathway analysis indicated marked enrichment in the ferroptosis signaling pathway (Fig. 2B–C). Subsequently, RAW264.7 cells were polarized into the M2 phenotype using IL-4 induction and then exposed to PM_2.5_ (Fig. 2D). Relative to the M2 group, the M2 + PM_2.5_ group displayed a marked reduction in mRNA and protein expression levels of M2 markers CD206 and Arg1 (Fig. 2E–F). Flow cytometry analysis further confirmed a reduction in the proportion of CD206^+^ cells (Fig. 2I). Concurrently, mRNA and protein levels of the ferroptosis-related markers ACSL4 and PTGS2 were considerably upregulated, while FTH1 and GPX4 expression levels were markedly downregulated (Fig. 2G–H). Intracellular Fe^2+^, Malondialdehyde (MDA), and lipid ROS levels were significantly increased, whereas Glutathione (GSH) content was decreased (Fig. 2J–L). Transmission electron microscopy identified classical ferroptotic ultrastructural alterations with disrupted cristae, elevated membrane density, and condensed volume. Notably, the outer mitochondrial membranes remained intact, and nuclear morphology displayed no apparent abnormalities (Fig. 2R–S). These results are consistent with observations in THP-1 cells (Fig. 2M–Q).

In a mouse model of PM_2.5_-aggravated asthma, PM_2.5_ exposure caused a marked elevation in the percentage of CD206^+^ACSL4^+^ cells in BALF and lung (Fig. 2T–U, S32A), elevated Fe^2+^ levels, and reduced GSH levels in BALF and serum (Fig. 2V, S12). Collectively, these findings confirm that PM_2.5_ exposure induces ferroptosis in M2 macrophages.

### 3.3 Ferroptosis inhibition restores M2 macrophage proportion and ameliorates asthma symptoms

**Fig. 3:**
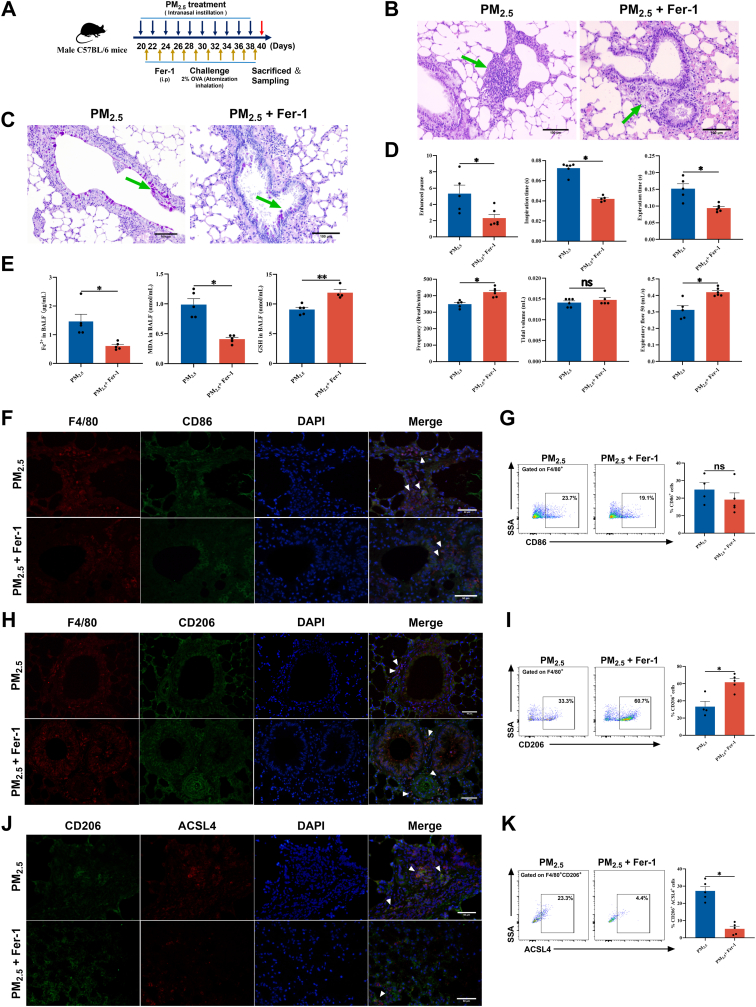
**The ferroptosis inhibitor Fer-1 restored the proportion of M2 macrophages *in vivo*. (A)** Schematic diagram of the Fer-1 intervention in a PM_2.5_-aggravated asthma mouse model. **(B)** Representative photomicrographs of H&E-stained lung tissue sections. Scale bar: 100 μm. Green arrows indicate infiltrating inflammatory cells. **(C)** Representative images of PAS-stained lung tissue sections. Scale bar: 100 μm. Green arrows denote secreted mucus and proliferated goblet cells. **(D)** Lung function of mice (n = 6). **(E)** Measurement of Fe^2+^, MDA, and GSH levels in mouse BALF (n = 6). **(F)** Immunofluorescence staining for F4/80 and CD86 in mouse lung tissue. Scale bar: 50 μm. **(G)** Flow cytometry analyzed the proportion of F4/80^+^CD86^+^ cells in mouse BALF (n = 6). **(H)** Immunofluorescence staining for F4/80 and CD206 in mouse lung tissue. Scale bar: 50 μm. **(I)** Flow cytometry was used to analyze the proportion of F4/80^+^CD206^+^ cells in mouse BALF (n = 6). **(J)** Immunofluorescence staining for CD206 and ACSL4 in mouse lung tissue. Scale bar: 50 μm. **(K)** Flow cytometry analysis of the proportion of CD206^+^ACSL4^+^ cells in mouse BALF (n = 6). The PM_2.5_ group data are illustrated in Fig. 3, Fig. 6, derived from the same set of independent experiments. Values are expressed as the means ± SD. The sign * indicates *P* < 0.05, while ** indicates *P* < 0.01, and *ns* indicates no significance.

Administration of the ferroptosis inhibitor Fer-1 in the PM_2.5_-aggravated murine asthma model significantly restored body weight (Fig. 3A, S2B) and concomitantly reduced the organ coefficients of lung (Fig. S13A). Histological evaluation by H&E and PAS staining demonstrated attenuated inflammatory cell infiltration, reduced mucus secretion, and inhibited goblet cell hyperplasia in the lung tissue (Fig. 3B–C, S15). Concurrently, lung function parameters were significantly improved (Fig. 3D). Biochemical analysis of BALF and serum exhibited a pronounced decrease in Fe^2+^ and MDA levels, along with a significant increase in GSH content (Fig. 3E, S16). Immunofluorescence staining and flow cytometry analysis further demonstrated that Fer-1 treatment notably increased the proportion of M2 macrophages in lung tissue and BALF, while reducing the frequency of CD206^+^ACSL4^+^ double-positive cells. Conversely, the population of M1 macrophages remained unchanged (Fig. 3F–K, S32B).

**Fig. 4:**
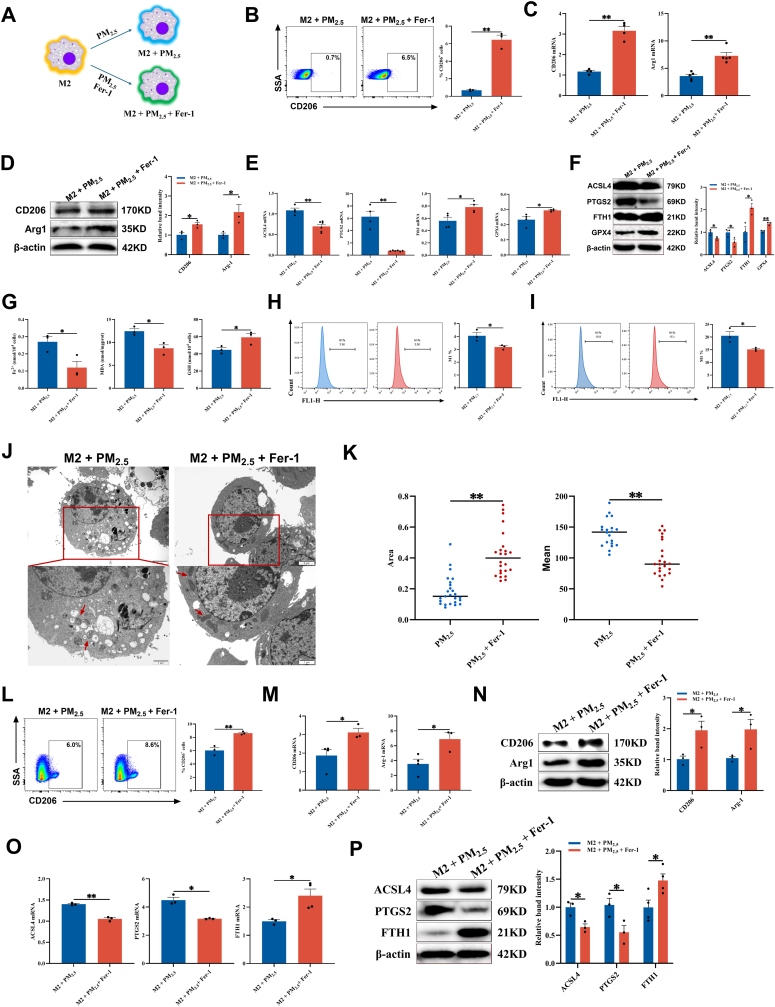
**The ferroptosis inhibitor Fer-1 restored the proportion of M2 macrophages *in vitro*. (A)** Schematic of Fer-1 intervening with M2 macrophages *in vitro*. **(B)** Flow cytometry analysis of the proportion of CD206^+^ cells in M2 macrophages derived from RAW264.7 cells that were treated with PM_2.5_ and Fer-1 (n = 3). **(C)** The mRNA levels of CD206 and Arg1 were measured by RT-qPCR in M2 macrophages derived from RAW264.7 cells treated with PM_2.5_ and Fer-1 (n = 5). **(D)** The protein levels of CD206 and Arg1 were detected by Western blot in M2 macrophages derived from RAW264.7 cells that were treated with PM_2.5_ and Fer-1 (n = 3). **(E)** RT-qPCR detected the mRNA levels of ACSL4, PTGS2, GPX4, and FTH1 in M2 macrophages derived from RAW264.7 cells that were treated with PM_2.5_ and Fer-1 (n = 6). **(F)** Protein levels of ACSL4, PTGS2, GPX4, and FTH1 were detected by Western blot in M2 macrophages derived from RAW264.7 cells treated with PM_2.5_ and Fer-1 (n = 3). **(G)** Measurement of Fe^2+^, MDA, and GSH levels in M2 macrophages derived from RAW264.7 cells that were treated with PM_2.5_ and Fer-1 (n = 3). **(H)** Cells were stained with FerroOrange and analyzed by flow cytometry (n = 3). **(I)** Cells were stained with BODIPY™ 581/591 C11 and analyzed by flow cytometry (n = 3). **(J)** Representative TEM images of M2 macrophages derived from RAW264.7 cells that were treated with PM_2.5_ and Fer-1. Scale bar: 2 μm. Red arrows indicate mitochondria. **(K)** Quantitative analysis of mitochondrial volume and density (n = 20–30). **(L)** Flow cytometry analysis was used to analyze the proportion of CD206^+^ cells in M2 macrophages derived from THP-1 cells that were treated with PM_2.5_ and Fer-1 (n = 3). **(M)** The mRNA levels of CD206 and Arg1 were measured by RT-qPCR in M2 macrophages derived from THP-1 cells treated with PM_2.5_ and Fer-1 (n = 4). **(N)** The protein levels of CD206 and Arg1 were detected by Western blot in macrophages derived from THP-1 cells that were treated with PM_2.5_ and Fer-1 (n = 3). **(O)** RT-qPCR detected the mRNA levels of ACSL4, PTGS2, and FTH1 in M2 macrophages derived from THP-1 cells that were treated with PM_2.5_ and Fer-1 (n = 4). **(P)** Protein levels of ACSL4, PTGS2, and FTH1 were detected by Western blot in M2 macrophages derived from THP-1 cells treated with PM_2.5_ and Fer-1 (n = 4). Values are expressed as the means ± SD. * indicates *P* < 0.05, ** indicates *P* < 0.01.

In the *in vitro* model of PM_2.5_-stimulated M2 macrophages, intervention with Fer-1 raised the proportion of CD206^+^ cells (Fig. 4A–B). A CCK-8 assay was performed to screen for a non-cytotoxic concentration of Fer-1. As illustrated in Fig. S17, Fer-1 at concentrations up to 10 μM did not affect cell viability. Consequently, 10 μM was used in subsequent experiments. Moreover, the CCK-8 assay demonstrated that PM_2.5_ treatment decreased RAW264.7 cell viability in a time-dependent manner, which was markedly reversed by Fer-1 co-treatment (Fig. S18). Both mRNA and protein expression of the M2 markers CD206 and Arg1 were markedly upregulated (Fig. 4C–D), whereas expression levels of ferroptosis-related markers ACSL4 and PTGS2 were significantly downregulated. Additionally, FTH1 and GPX4 expression levels were notably elevated (Fig. 4E–F). Intracellular Fe^2+^, MDA, and lipid ROS levels were significantly reduced, while GSH content was increased (Fig. 4G–I). Mitochondrial morphology also exhibited substantial recovery, characterized by restored cristae structure and reduced condensation (Fig. 4J–K). It has also been demonstrated in THP-1 cells (Fig. 4L–P). These findings indicate that Fer-1 alleviates PM_2.5_-aggravated asthma by inhibiting ferroptosis in M2 macrophages and restoring their population and functional phenotype.

### 3.4 Activation of the Fra2/LCN2 axis in M2 macrophages mediates PM_2.5_-induced asthma exacerbation

**Fig. 5:**
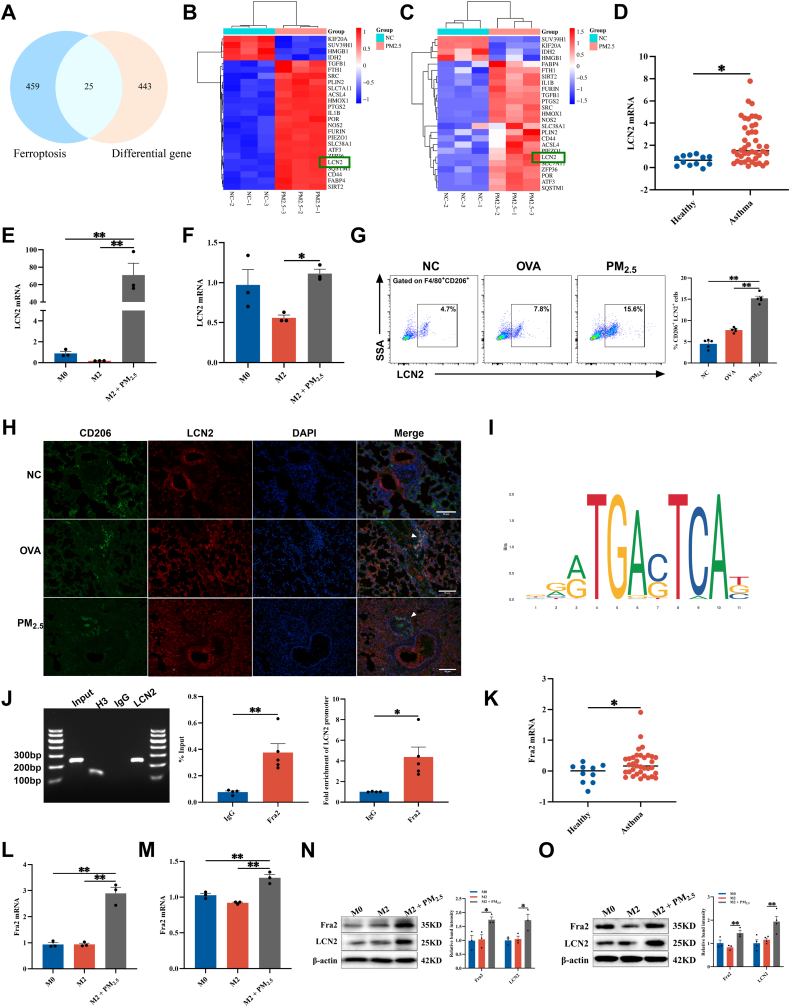
PM_2.5_ activates the Fra2/LCN2 axis. (A) Venn diagram of 468 DEGs and ferroptosis pathway genes from the FerrDb V2 database. **(B)** Clustering heatmap of transcriptome data for 25 ferroptosis-related DEGs. **(C)** Clustering heatmap of proteomic data for 25 ferroptosis-related DEGs. **(D)** The level of LCN2 in the GSE74986 dataset (n = 12–44). **(E)** LCN2 mRNA levels were measured by RT-qPCR in PM_2.5_-stimulated M2 macrophages derived from RAW264.7 cells (n = 3). **(F)** LCN2 mRNA levels were measured by RT-qPCR in PM_2.5_-stimulated M2 macrophages derived from THP-1 cells (n = 3). **(G)** Flow cytometry quantified the proportion of CD206^+^LCN2^+^ cells in mouse BALF (n = 6). **(H)** Immunofluorescence staining for CD206 and LCN2 in mouse lung tissue. Scale bar: 50 μm. **(I)** The corresponding motif logo of Fra2. **(J)** ChIP-qPCR indicated that the Fra2 protein specifically bound to the LN2 promoter region in RAW264.7 cells (n = 5). **(K)** The level of Fra2 in the GSE74986 dataset (n = 10-35). **(L)** Fra2 mRNA levels were measured by RT-qPCR in PM_2.5_-stimulated M2 macrophages derived from RAW264.7 cells (n = 3). **(M)** Fra2 mRNA levels were measured by RT-qPCR in PM_2.5_-stimulated M2 macrophages derived from THP-1 cells (n = 3). **(N)** The protein levels of Fra2 and LCN2 were detected by Western blot in PM_2.5_-stimulated M2 macrophages derived from RAW264.7 cells (n = 3). **(O)** The protein levels of Fra2 and LCN2 were detected by Western blot in PM_2.5_-stimulated M2 macrophages derived from THP-1 cells (n = 3). Values are expressed as the means ± SD. * indicates *P* < 0.05, ** indicates *P* < 0.01.

Since 468 DEGs were abundant in the ferroptosis signaling pathway, we overlapped these DEGs with ferroptosis-related genes obtained from the FerrDb (version 2) database, identifying 25 key ferroptosis-associated DEGs (Fig. 5A). Cluster analysis of transcriptomic and proteomic data for these 25 DEGs revealed that LCN2 expression was substantially elevated at both the transcriptional and translational levels (*P* < 0.01), exhibiting the highest log_2_FC among all DEGs (Fig. 5B–C). Furthermore, analysis of the publicly available Gene Expression Omnibus (GEO) dataset GSE74986, which profiles DEGs in BALF cells from patients with asthma and healthy controls, indicated that LCN2 expression was strikingly elevated in patients with asthma (Fig. 5D). Similarly, in M2 macrophages derived from RAW264.7 and THP-1 cells, LCN2 expression was markedly increased after PM_2.5_ stimulations (Fig. 5E–F, N-O). In a mouse model of PM_2.5_-aggravated asthma, flow cytometry analysis demonstrated a remarkable increase in the proportion of CD206^+^LCN2^+^ cells in BALF, along with enhanced colocalization of CD206 and LCN2 in lung tissues (Fig. 5G–H, S32A). These findings indicate that PM_2.5_ induces upregulation of LCN2 expression in M2 macrophages under asthmatic conditions.

Subsequently, we utilized the AnimalTFDB (version 4.0) database to predict potential TFs regulating LCN2 and intersected these with the previously identified 468 DEGs, yielding four candidate TFs: Atf3, Fra2, Sp100, and Nfκb1. Prediction of TF binding sites within the LCN2 promoter was conducted using the JASPAR database (a database of transcription factor binding profiles). Considering both binding scores and the number of binding sites, Fra2 was selected as a specific regulator of LCN2 (Fig. 5I). To determine whether Fra2 directly regulates LCN2 transcription, we performed ChIP-qPCR. The chromatin fragments precipitated by Fra2-specific antibody indicated significant enrichment at the LCN2 promoter region compared to the non-specific IgG control, with an enrichment fold change greater than 4 (*P* < 0.05, n = 3), confirming that Fra2 specifically binds to the LCN2 promoter (Fig. 5J). Moreover, in the GSE74986 dataset, Fra2 expression was evidently higher in BALF cells from patients with asthma than in healthy controls (Fig. 5K). Consistent results were observed in PM_2.5_-stimulated M2 macrophage models (Fig. 5L–O). We also compared the effects of PM_2.5_ on ferroptosis and Fra2/LCN2 axis activation between M1 and M2 macrophages. As illustrated in Figs. S19–20, PM_2.5_ exposure failed to significantly induce ferroptosis in M1 macrophages, whereas the upregulation of Fra2 and LCN2 expression was significantly more pronounced in M2 cells than in M1 cells. These findings demonstrate that M2 macrophages are more sensitive than M1 macrophages to PM_2.5_-induced ferroptosis and Fra2/LCN2 axis activation. Collectively, these results suggest that PM_2.5_ exacerbates asthma by activating the Fra2/LCN2 signaling axis in M2 macrophages.

### 3.5 Targeting the Fra2/LCN2 axis attenuates ferroptosis and alleviates asthma

**Fig. 6:**
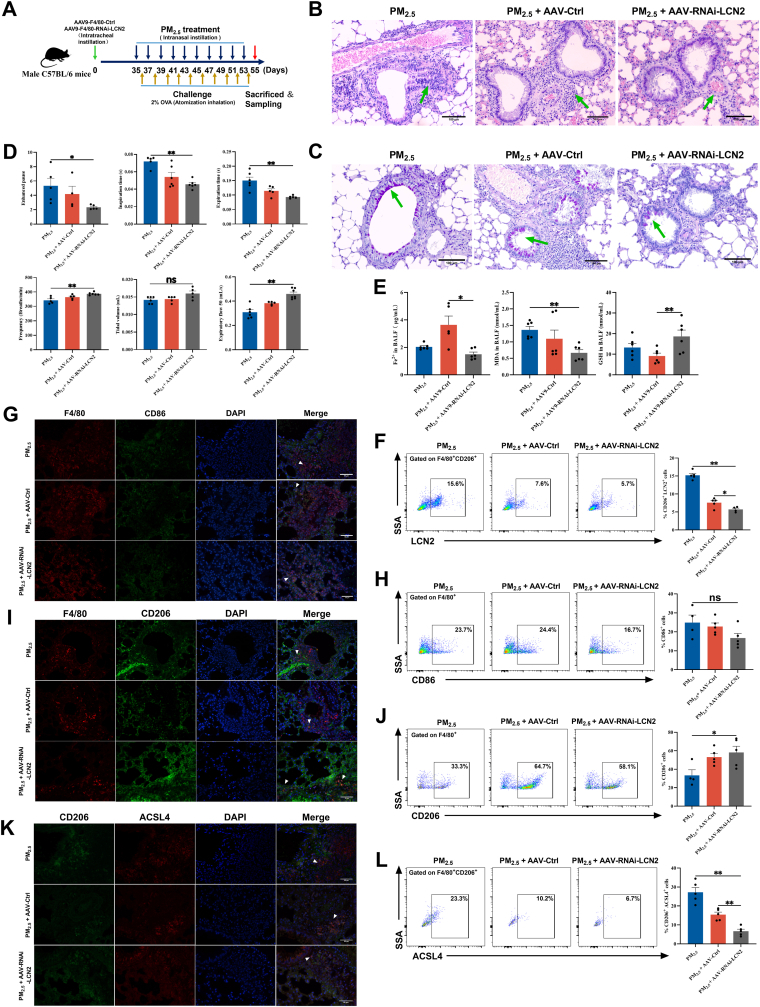
**AAV9-mediated macrophage-specific LCN2 knockdown attenuates asthma progression by suppressing ferroptosis in mice. (A)** Schematic diagram of inducing PM_2.5_-aggravated asthma mouse model after AAV9-mediated macrophage-specific LCN2 knockdown. **(B)** Representative photomicrographs of H&E-stained lung tissue sections. Scale bar: 100 μm. Green arrows indicate infiltrating inflammatory cells. **(C)** Representative images of PAS-stained lung tissue sections. Scale bar: 100 μm. Green arrows denote secreted mucus and proliferated goblet cells. **(D)** Lung function of mice (n = 6). **(E)** Measurement of Fe^2+^, MDA, and GSH levels in mouse BALF (n = 6). **(F)** Flow cytometry analysis of the proportion of CD206^+^LCN2^+^ cells in mouse BALF (n = 5). **(G)** Immunofluorescence staining for F4/80 and CD86 in mouse lung tissue. Scale bar: 50 μm. **(H)** Flow cytometry analysis of the proportion of F4/80^+^CD86^+^ cells in mouse BALF (n = 5). **(I)** Immunofluorescence staining for F4/80 and CD206 in mouse lung tissue. Scale bar: 50 μm. **(J)** Flow cytometry analysis of the proportion of F4/80^+^CD206^+^ cells in mouse BALF (n = 5). **(K)** Immunofluorescence staining for CD206 and ACSL4 in mouse lung tissue. Scale bar: 50 μm. **(L)** Flow cytometry analysis of the proportion of CD206^+^ACSL4^+^ cells in mouse BALF (n = 5). The PM_2.5_ group data are illustrated in Fig. 3, Fig. 6 are derived from the same set of independent experiments. Values are expressed as the means ± SD. * indicates *P* < 0.05, ** indicates *P* < 0.01 and ns for no significance.

To investigate whether macrophage-specific LCN2 deficiency modulates PM_2.5_-induced exacerbation of asthma, we generated mice with conditional knockdown of LCN2 in macrophages via intratracheal injection of adeno-associated virus 9 (AAV9) carrying F4/80 promoter-driven siRNA targeting LCN2 [AAV9-F4/80-siRNA-LCN2-Green Fluorescent Protein (GFP) ]or a non-targeting control siRNA (AAV9-F4/80-siRNA-Ctrl-GFP), respectively (Fig. 6A). Successful infection was confirmed by detecting GFP fluorescence in BALF (Fig. S21). Effective knockdown of LCN2 in macrophages was validated by flow cytometric analysis of F4/80^+^LCN2^+^ cells in BALF (Fig. S22).

Macrophage-specific knockdown of LCN2 resulted in a pronounced increase in body weight (Fig. S2C), a decrease in the organ-to-body weight ratios of the heart, liver, and lung (Fig. S25), and a reduction in serum OVA-sIgE levels (Fig. S4C), along with markedly attenuated inflammatory cell infiltration, mucus hypersecretion, and goblet cell hyperplasia in lung tissue (Fig. 6B–C, S28). Furthermore, LCN2 knockdown significantly reduced total cell counts and the percentage of lymphocytes in BALF, while increasing the percentage of macrophages (Fig. S26). Compared with PM_2.5_-exposed asthmatic mice, those treated with AAV9-F4/80-siRNA-LCN2 exhibited significantly improved lung function (Fig. 6D), a reduced proportion of CD206^+^LCN2^+^ cells in BALF (Fig. 6F), as well as decreased levels of Fe^2+^ and MDA in both BALF and serum, alongside increased GSH levels (Fig. 6E, S27). Immunofluorescence staining and flow cytometry analysis further demonstrated a significant rise in the proportion of M2 macrophages in lung tissue and BALF, which was accompanied by a decrease in CD206^+^ACSL4^+^ cells. Conversely, the proportion of M1 macrophages remained unchanged (Fig. 6G–L, S32C). Collectively, these results indicate that macrophage-specific knockdown of LCN2 ameliorates PM_2.5_-aggravated asthma by reducing the population of M2 macrophages undergoing ferroptosis.

**Fig. 7:**
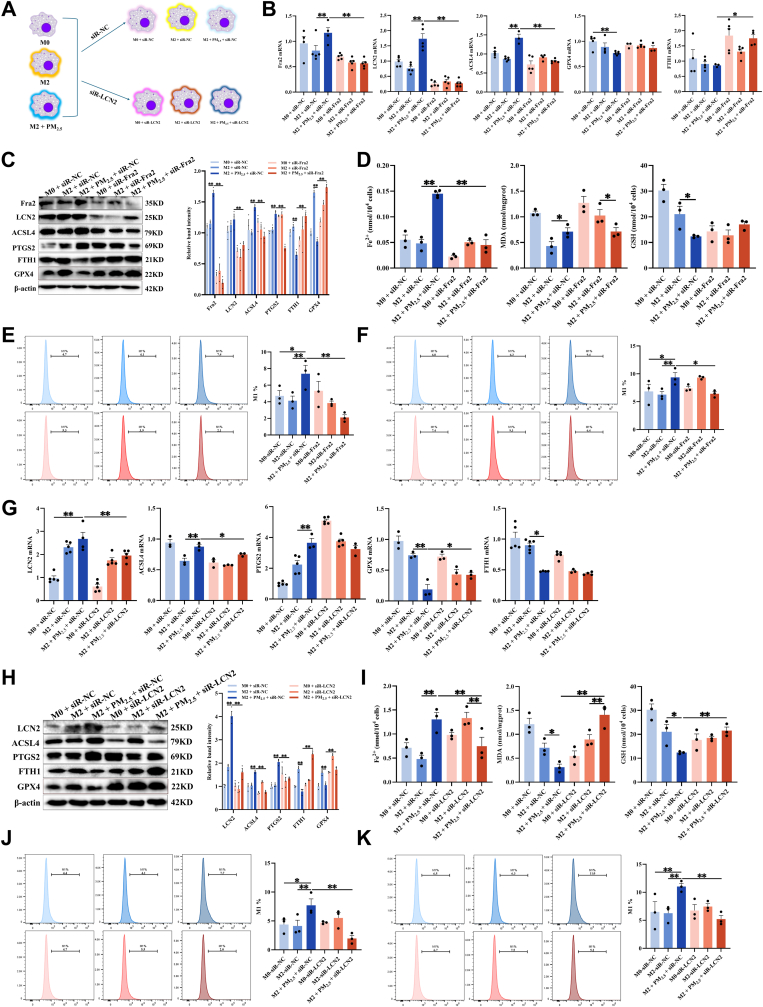
**Fra2/LCN2 axis inhibition reduces ferroptosis in M2 macrophages. (A)** Schematic of siRNA-Fra2/LCN2 intervening with M2 macrophages *in vitro*. **(B)** The mRNA levels of Fra2, LCN2, ACSL4, GPX4 and FTH1 were measured by RT‒qPCR in siR-Fra2-treated RAW264.7 cells-derived M2 macrophages (n = 5). **(C)** The protein levels of Fra2, LCN2, ACSL4, PTGS2, GPX4 and FTH1 were measured by Western blot in siR-Fra2-treated RAW264.7 cells-derived M2 macrophages (n = 3). **(D)** Measurement of Fe^2+^, MDA, and GSH levels in siR-Fra2-treated RAW264.7 cells-derived M2 macrophages (n = 3). **(E)** Cells were stained with FerroOrange and analyzed by flow cytometry (n = 3). **(F)** Cells were stained with BODIPY™ 581/591 C11 and analyzed by flow cytometry (n = 3). **(G)** The mRNA levels of LCN2, ACSL4, PTGS2, GPX4 and FTH1 detected by RT-qPCR in siR-LCN2-treated RAW264.7 cells-derived M2 macrophages (n = 3–6). **(H)** The protein levels of LCN2, ACSL4, PTGS2, GPX4 and FTH1 were measured by Western blot in siR-LCN2-treated RAW264.7 cells-derived M2 macrophages (n = 3). **(I)** Measurement of Fe^2+^, MDA, and GSH levels in siR-LCN2-treated RAW264.7 cells-derived M2 macrophages (n = 3). **(J)** Cells were stained with FerroOrange and analyzed by flow cytometry (n = 3). **(K)** Cells were stained with BODIPY™ 581/591 C11 and analyzed by flow cytometry (n = 3). Values are expressed as the means ± SD. * indicates *P* < 0.05, ** indicates *P* < 0.01.

For the *in vitro* experiments, we transfected M2 macrophages with siRNA targeting Fra2 or LCN2, then exposed them to PM_2.5_ (Fig. 7A, S29). As illustrated in Fig. 7B–C, knockdown of Fra2 significantly reduced LCN2 expression levels. Furthermore, Fra2 silencing led to marked decreases in Fe^2+^, MDA, lipid ROS, ACSL4, and PTGS2 levels, while elevating GPX4 and FTH1 expression (Fig. 7B–F). Similarly, knockdown of LCN2 also resulted in reduced levels of Fe^2+^, MDA, lipid ROS, ACSL4, and PTGS2, along with increased expression of GSH, GPX4, and FTH1 (Fig. 7G–K). These results indicate that suppressing the Fra2/LCN2 signaling axis effectively mitigates ferroptosis in M2 macrophages and promotes their functional presence.

### 3.6 PM_2.5_ suppresses mitophagy to promote ferroptosis in M2 macrophages

**Fig. 8:**
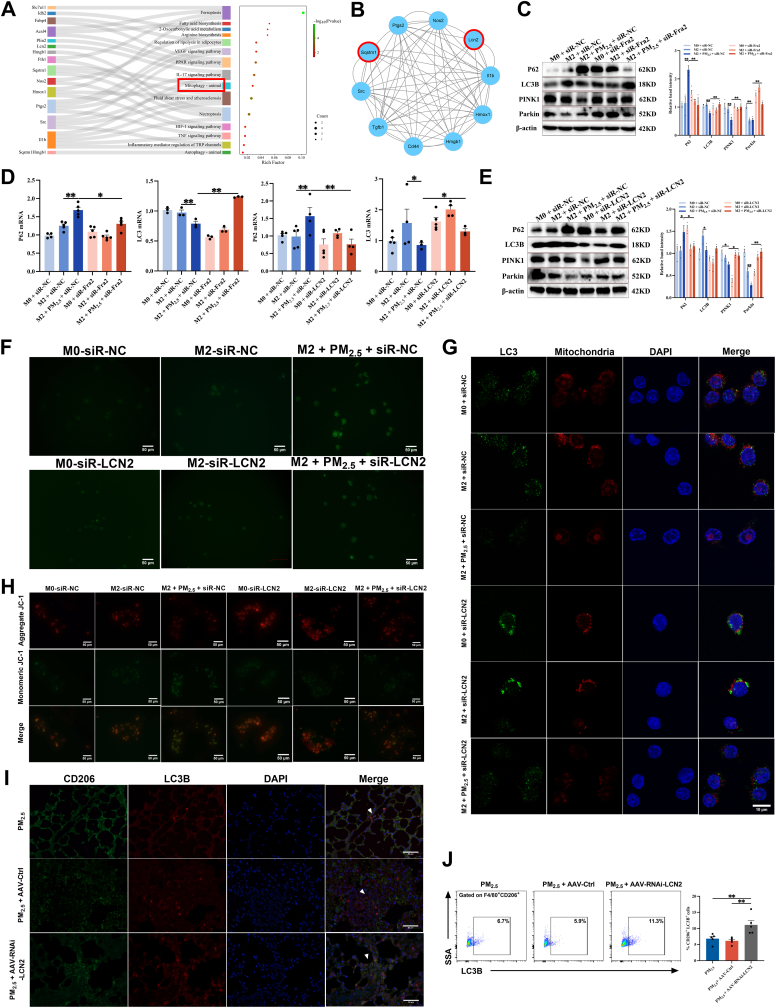
**PM_2.5_ suppresses mitophagy to promote ferroptosis in M2 Macrophages. (A)** KEGG pathway enrichment analysis of the 25 ferroptosis-related DEGs. **(B)** PPI network of hub genes. **(C)** The protein levels of P62, LC3B, PINK1, and Parkin were measured by Western blot in siR-Fra2-treated RAW264.7 cells-derived M2 macrophages (n = 3). **(D)** RT-qPCR detected the mRNA levels of P62 and LC3B in siR-Fra2/LCN2-treated RAW264.7 cells-derived M2 macrophages (n = 5). **(E)** The protein levels of P62, LC3B, PINK1, and Parkin were measured by Western blot in siR-LCN2-treated RAW264.7 cells-derived M2 macrophages (n = 3). **(F)** Representative fluorescence images of ROS in siR-LCN2-treated RAW264.7 cells-derived M2 macrophages. Scale bar: 50 μm. **(G)** Representative fluorescence images of LC3B and mitochondrial colocalization in siR-LCN2-treated RAW264.7 cells-derived M2 macrophages. Scale bar: 10 μm. **(H)** Representative fluorescence images indicating mitochondrial membrane potential (ΔΨm) in siR-LCN2-treated RAW264.7 cells-derived M2 macrophages using JC-1 staining. Scale bar: 50 μm. **(I)** Immunofluorescence staining for CD206 and LC3B in mouse lung tissue. Scale bar: 50 μm. **(J)** Flow cytometry analysis was used to analyze the proportion of CD206^+^LC3B^+^ cells in mouse BALF (n = 5). Values are expressed as the means ± SD. * indicates *P* < 0.05, ** indicates *P* < 0.01.

To investigate the specific molecular mechanism through which the Fra2/LCN2 signaling axis regulates ferroptosis in M2 macrophages, we performed KEGG pathway enrichment and protein-protein interaction (PPI) network analyses on 25 previously identified DEGs. The term “mitochondrial autophagy” was significantly enriched (Fig. 8A–B, S30). We subsequently examined the expression levels of P62, LC3B, PINK1, and Parkin in M2 macrophages. We found that PM_2.5_ exposures upregulated P62 expression; however, they downregulated LC3B, PINK1, and Parkin expression. Compared with the siR-NC group, knockdown of Fra2/LCN2 reduced P62 levels and elevated LC3B, PINK1, and Parkin levels (Fig. 8C–E). Furthermore, PM_2.5_ exposures increased ROS production, reduced mitochondrial membrane potential, and diminished mitochondrial-LC3B colocalization in M2 macrophages. Knockdown of LCN2 markedly reversed these effects (Fig. 8F–H, S32D–F). In asthmatic mice with macrophage-specific LCN2 knockdown exposed to PM_2.5_, the proportion of CD206^+^LC3B^+^ cells in lung tissue and BALF was markedly reduced compared to the PM_2.5_-exposed group (Fig. 8I–J, S32C). These results indicate that PM_2.5_ exposure suppresses mitophagy in M2 macrophages, whereas inhibition of the Fra2/LCN2 signaling axis restores mitochondrial autophagy function.

### 3.7 Mitophagy inhibition mediates Fra2/LCN2 axis-induced ferroptosis

To investigate whether the Fra2/LCN2 axis promotes ferroptosis by inhibiting mitophagy, we performed gain-of-function and pharmacological intervention experiments in PM_2.5_-exposed M2 macrophages.

**Fig. 9:**
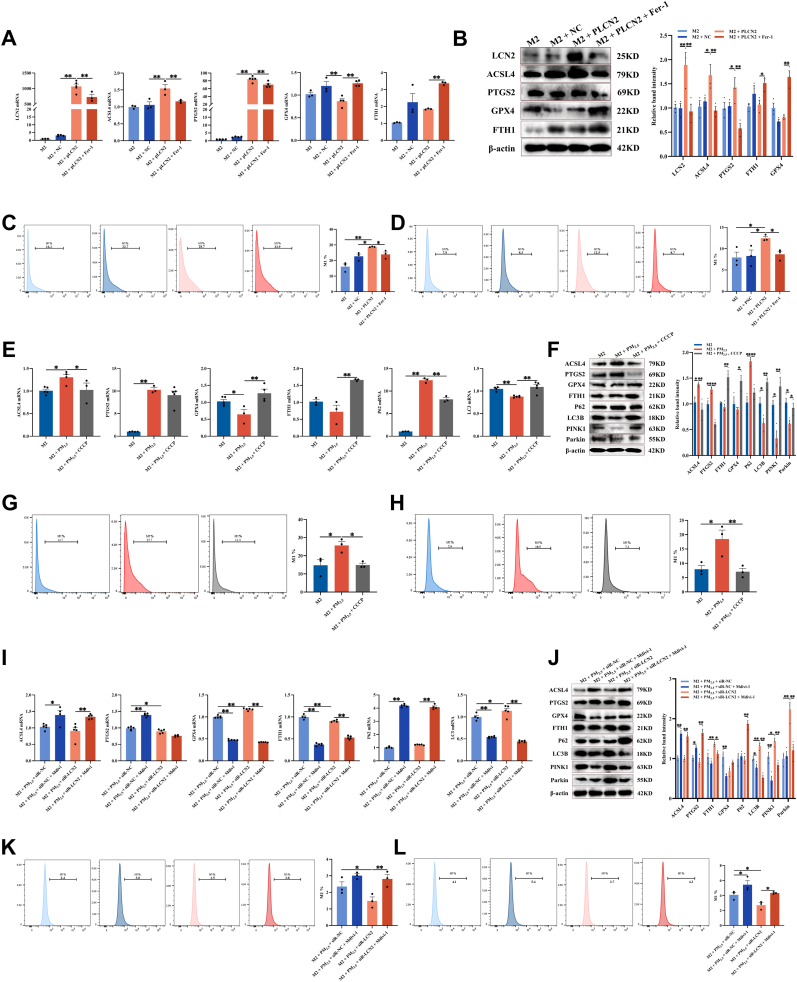
**Mitophagy inhibition mediates Fra2/LCN2 axis-induced ferroptosis. (A)** The mRNA levels of LCN2, ACSL4, PTGS2, GPX4 and FTH1 detected by RT-qPCR in pLCN2-treated RAW264.7 cells-derived M2 macrophages (n = 5). **(B)** The protein levels of LCN2, ACSL4, PTGS2, GPX4 and FTH1 detected by Western blot in pLCN2-treated RAW264.7 cells-derived M2 macrophages (n = 3). **(C)** Flow cytometric analysis of intracellular Fe^2+^ levels detected by FerroOrange staining in pLCN2-treated RAW264.7 cells-derived M2 macrophages (n = 3). **(D)** Flow cytometric analysis of intracellular lipid peroxidation levels detected by BODIPY™ 581/591 C11 staining in pLCN2-treated RAW264.7 cells-derived M2 macrophages (n = 3). **(E)** The mRNA levels of ACSL4, PTGS2, GPX4, FTH1, P62, and LC3 detected by RT-qPCR in CCCP-treated RAW264.7 cells-derived M2 macrophages (n = 5). **(F)** The protein levels of ACSL4, PTGS2, GPX4, FTH1, P62, LC3B, PINK1, and Parkin detected by Western blot in CCCP-treated RAW264.7 cells-derived M2 macrophages (n = 3). **(G)** Flow cytometric analysis of intracellular Fe^2+^ levels detected by FerroOrange staining in CCCP-treated RAW264.7 cells-derived M2 macrophages (n = 3). **(H)** Flow cytometric analysis of intracellular lipid peroxidation levels detected by BODIPY™ 581/591 C11 staining in CCCP-treated RAW264.7 cells-derived M2 macrophages (n = 3). **(I)** The mRNA levels of ACSL4, PTGS2, GPX4, FTH1, P62 and LC3 detected by RT-qPCR in Mdivi-1-treated RAW264.7 cells-derived M2 macrophages (n = 5). **(J)** The protein levels of ACSL4, PTGS2, GPX4, FTH1, P62, LC3B, PINK1 and Parkin detected by Western blot in Mdivi-1-treated RAW264.7 cells-derived M2 macrophages (n = 3). **(K)** Flow cytometric analysis of intracellular Fe^2+^ levels detected by FerroOrange staining in Mdivi-1-treated RAW264.7 cells-derived M2 macrophages (n = 3). **(L)** Flow cytometric analysis of intracellular lipid peroxidation levels detected by BODIPY™ 581/591 C11 staining in Mdivi-1-treated RAW264.7 cells-derived M2 macrophages (n = 3). Values are expressed as the means ± SD. * indicates *P* < 0.05, ** indicates *P* < 0.01.

First, we overexpressed LCN2 in M2 macrophages using an LCN2 plasmid. As illustrated in Fig. 9A–D, LCN2 overexpression recapitulated the effects of PM_2.5_ exposure, as evidenced by increased intracellular iron levels, elevated lipid peroxidation, upregulation of ACSL4 and PTGS2 expression, and downregulation of GPX4 and FTH2 expression, confirming the induction of ferroptosis. These results indicate that LCN2 overexpression alone is sufficient to trigger ferroptosis in M2 macrophages.

Next, we treated PM_2.5_-stimulated M2 macrophages with Carbonyl Cyanide m-Chlorophenyl Hydrazone (CCCP), a known inducer of mitophagy (Fig. S31A). The results indicated that CCCP treatment significantly restored PINK1 and Parkin expression, increased LC3B levels, and decreased p62 levels. Importantly, CCCP treatment also attenuated PM_2.5_-induced ferroptosis, as demonstrated by reduced iron accumulation, decreased lipid peroxidation, and restoration of ACSL4, PTGS2, GPX4, and FTH1 expression (Fig. 9E–H). Conversely, treatment with Mdivi-1, a specific mitophagy inhibitor (Fig. S31B), further exacerbated PM_2.5_-induced impairment of mitophagy and ferroptosis. Notably, when Mdivi-1 was combined with si-LCN2, the protective effect of si-LCN2 was substantially diminished (Fig. 9I–L). Collectively, these findings indicate that inhibition of mitophagy by the Fra2/LCN2 axis is a key upstream event in PM_2.5_-induced ferroptosis in M2 macrophages; restoring mitophagy or knocking down LCN2 effectively alleviates ferroptosis, suggesting this axis as a potential therapeutic target.

## 4 Discussion

Asthma is a prevalent chronic inflammatory airway disease, the pathogenesis of which is strongly influenced by environmental factors. It is driven by a diverse array of immune cells, including eosinophils, basophils, neutrophils, mast cells, macrophages, epithelial cells, and T lymphocytes. Our previous work has demonstrated that PM_2.5_ exposure disrupts the Th1/Th2 and Th17/Treg balance, promoting asthma exacerbation [27,28]. As innate immune cells, macrophages are fundamental to immune surveillance within the asthmatic lung and facilitate the development of innate and adaptive immune responses. The deposition of PM_2.5_ particles in the distal airways and alveolar regions enables their interaction with resident alveolar macrophages. Ultrafine particles (<0.1 μm) can cross the alveolar barrier, enter the systemic circulation, and subsequently induce serious health effects in secondary organs and tissues [29]. Our collective findings demonstrate that PM_2.5_ induces a marked imbalance between M1 and M2 macrophages primarily by triggering a specific form of programmed cell death—ferroptosis—in M2 macrophages. Moreover, we have identified the Fra2/LCN2 signaling axis as the critical molecular pathway driving this process and uncovered impaired mitophagy as a key upstream event leading to ferroptotic cell death in M2 macrophages. This multi-layered mechanism provides a new conceptual framework for understanding environmental asthma exacerbation and reveals several potential therapeutic targets.

As a major immune cell population in the respiratory tract, macrophages polarize into pro-inflammatory M1 or anti-inflammatory M2 phenotypes. An imbalance in M1/M2 macrophage polarization can have detrimental consequences, contributing to a range of inflammatory processes and disease pathologies [30]. In asthma, macrophages are recruited to the airway tissues and bronchial epithelium, where they release elevated levels of inflammatory mediators [Tumor Necrosis Factor (TNF)-α and Interleukin (IL)-1β] that perpetuate chronic airway inflammation [31]. In contrast to their pro-inflammatory role, the tissue-reparative function of macrophages involves the secretion of anti-inflammatory mediators [IL-10 and Transforming Growth Factor (TGF-β) ] and the phagocytic clearance of apoptotic cells [32]. Consequently, modulating macrophage polarization to suppress excessive inflammation and enhance anti-inflammatory responses represents a promising therapeutic strategy for asthma. Both *in vivo* and *in vitro* experiments in this study demonstrated that PM_2.5_ exposure alters macrophage polarization, increasing the M1 subset and decreasing the M2 subset, thereby exacerbating asthma symptoms. These findings are consistent with earlier reports that PM_2.5_ promotes M1 polarization via ROS activation and suppresses M2 polarization through an mechanistic Target of Rapamycin (mTOR)-dependent pathway [33]. However, while prior research has largely focused on M1 activation, the mechanisms underlying PM_2.5_-induced inhibition of M2 macrophages remain elusive.

Ferroptosis is a form of regulated cell death characterized by iron dependence and excessive lipid peroxidation [34]. Growing evidence suggests that a significant link exists between ferroptosis and the development of asthma [35,36]. Our study reveals that exposure to PM_2.5_ increases levels of MDA and Fe^2+^, both key ferroptosis-related markers, in the serum and BALF of OVA-induced asthmatic mice, and decreases GSH. Administration of a ferroptosis inhibitor reversed these PM_2.5_-induced changes. The inhibitor also ameliorated PM_2.5_-induced impairment of lung function, inflammatory cell infiltration, and mucus hypersecretion. These results confirm the earlier observations by Yi Z et al., who found that PM_2.5_ aggravates airway inflammation and mucus secretion in an House Dust Mite (HDM)-sensitized asthma model, and that Fer-1 treatment effectively attenuated these pathological features [37]. A study reveals that PM triggers ferroptosis in macrophages by upregulating key ferroptosis-related proteins, driving iron accumulation and lipid peroxidation [18]. A key finding from Liu et al. was the greater sensitivity of anti-inflammatory M2 macrophages to iron-induced ferroptosis compared to their pro-inflammatory M1 counterparts [38]. Consistent with these findings, another study demonstrated that M2 macrophages exhibit heightened susceptibility to RAS-Selective Lethal Compound 3 (RSL3)-induced ferroptosis relative to M1 macrophages [39]. In this study, PM_2.5_ exposures significantly increased the prevalence of ferroptotic M2 macrophages in the BALF of asthmatic mice. Corresponding *in vitro* experiments demonstrated that PM_2.5_ triggered dysregulation of ferroptosis-related proteins, elevated Fe^2+^, altered peroxidase activity, and induced mitochondrial abnormalities in M2 macrophages, collectively promoting robust ferroptosis and a subsequent decrease in M2 macrophage viability. Administration of the ferroptosis inhibitor Fer-1 markedly ameliorated these effects. A recent study demonstrated that SUMO-Specific Peptidase 3 (SENP3) promotes ferroptosis in M2 macrophages, thereby diminishing their numbers *in vivo*. SENP3 knockdown in macrophages was demonstrated to promote the M2 macrophage population and enhance diabetic wound healing. These findings are consistent with our conclusions [40]. Our rescue experiments utilizing ferroptosis inhibitors further substantiated the fundamental importance of ferroptosis in PM_2.5_-induced asthma exacerbation. Specific inhibition of ferroptosis restored M2 macrophage populations and significantly ameliorated core asthmatic features, including airway inflammation, mucus hypersecretion, and impaired lung function in murine models. These results provide direct causal evidence for the driving role of M2 macrophage ferroptosis in PM_2.5_-aggravated asthma. Consequently, ferroptosis inhibition holds promise as a viable strategy to alleviate the health impacts of air pollution on asthma-prone populations.

To elucidate the molecular mechanisms underlying PM_2.5_-induced ferroptosis in M2 macrophages, we identified the key ferroptosis-related gene LCN2 and its specific transcriptional regulator Fra2 through integrated multi-omics analysis. Experimental validation confirmed that Fra2 and LCN2 were consistently upregulated in BALF from patients with asthma (whole-cell transcriptome without cell-type resolution), in a mouse model of PM_2.5_-exacerbated asthma, and in an *in vitro* M2 macrophage model. Although the human data are limited to the bulk BALF level and lack cell-type specificity, the observed upregulation is consistent across *in vivo* and *in vitro* experimental systems. LCN2, commonly referred to as NGAL, was preliminarily purified from human neutrophils and functions as a pivotal iron transporter under both physiological and inflammatory conditions [41]. It facilitates cellular iron uptake by binding iron-carrying molecules such as ferritin and transferrin [42]. Higher levels of LCN2 have been found in the plasma of patients with asthma [43]. In OVA-induced asthmatic mice, LCN2 knockdown ameliorates inflammatory responses and restores the Treg/Th17 balance [44]. Fra2, a TF belonging to the Fos family, serves as a crucial component of the AP-1 TF complex and plays a significant role in the pathogenesis of chronic airway diseases, including asthma, pulmonary fibrosis, and chronic obstructive pulmonary disease [21]. Both patients with asthma and murine asthma models exhibit high Fra2 expression [45]. Our experimental results demonstrate that knockdown of either Fra2 or LCN2 via siRNA in PM_2.5_-stimulated M2 macrophages markedly ameliorates ferroptosis-related phenotypes. In a murine asthma model, macrophage-specific knockdown of LCN2 using AAV9 vectors significantly alleviated asthma symptoms, inhibited ferroptosis in M2 macrophages, and increased the percentage of M2 macrophages in bronchoalveolar lavage fluid. Previous studies indicate that LCN2 promotes acute lung injury by upregulating iron accumulation in macrophages [46]. Additionally, LCN2 knockdown has been demonstrated to protect against LPS-induced acute respiratory distress syndrome by blocking the Mitogen-Activated Protein Kinase (MAPK)/Extracellular Signal-Regulated Kinase (ERK) pathway and reducing ferroptosis-associated inflammation and oxidative stress [47]. Collectively, these findings suggest that PM_2.5_ upregulates Fra2, which transcriptionally activates LCN2 expression in M2 macrophages. This initiates iron homeostasis dysregulation, propagates excessive lipid peroxidation, and culminates in ferroptosis. Intervention studies confirm the functional importance of this pathway: inhibiting either Fra2 or LCN2 effectively protects M2 macrophages from ferroptosis, helps maintain their population, and attenuates asthma symptoms. Consequently, the Fra2/LCN2 axis represents a promising therapeutic target. Selective inhibition of ferroptosis in M2 macrophages may offer a novel treatment strategy for PM_2.5_-aggravated asthma.

A growing body of evidence demonstrates that ferroptosis and mitophagy are intricately linked, with mitophagy acting as a critical checkpoint in the ferroptosis pathway [48,49]. As a selective form of autophagy, mitophagy facilitates the clearance of damaged mitochondria, safeguards against mitochondrial dysfunction, and preserves normal mitochondrial morphology and function [50]. Defective mitophagy creates a permissive microenvironment for ferroptosis through ROS-mediated mitochondrial damage [51]. In this study, KEGG pathway enrichment analysis of 25 ferroptosis-related DEGs identified mitophagy as a significantly enriched pathway, with both protein and gene expression levels of Sequestosome 1 (SQSTM1)/p62 being markedly elevated. Previous studies have confirmed that cadmium exposure can inhibit mitophagy by inducing abnormal accumulation of SQSTM1/p62 [52]. Data from the current study demonstrated that PM_2.5_ stimulation elevated ROS levels, decreased mitochondrial membrane potential, significantly reduced LC3B production, and upregulated SQSTM1/p62 in M2 macrophages. These effects were effectively reversed by knocking down Fra2 or LCN2. Recent research has indicated that upregulated LCN2 in retinal pigment epithelial cells can form a complex with ATG4B and LC3, leading to lysosomal dysfunction, autophagy inhibition, abnormal iron accumulation, and ultimately ferroptosis [53]. Another study reported that FBXO2 activates PINK1-Parkin-mediated mitophagy and ubiquitinates LCN2 to suppress ferroptosis, thereby alleviating intervertebral disc degeneration [54]. Collectively, these dates indicate that PM_2.5_ exposure activates the TF Fra2, leading to upregulated transcription and expression of LCN2. This process inhibits mitophagy, promotes iron accumulation, and induces ferroptosis in M2 macrophages, thereby disrupting the M1/M2 macrophage balance and contributing to asthma exacerbation. These results provide novel mechanistic insights into how PM_2.5_ exacerbates asthma and suggest potential therapeutic targets.

Furthermore, there are several limitations regarding human relevance. First, BALF transcriptomic data from patients with asthma lack cell-type resolution and cannot be directly attributed to M2 macrophages. Direct evidence for PM_2.5_-induced ferroptosis in human macrophages is currently limited to THP-1 cell lines without validation in primary human macrophages. Consequently, our conclusions regarding the Fra2/LCN2 axis and ferroptosis pathway in humans should be considered exploratory, and future systematic validation using human primary macrophages is required. Second, regarding the animal model, this study employed an OVA-induced, PM_2.5_-exposed asthma mouse model that recapitulates key phenotypic features of allergic airway inflammation and PM_2.5_ exacerbations. However, the PM_2.5_ exposure protocol was short-term, whereas human exposure to air pollution is typically chronic and low-dose. Future studies should consider adopting chronic, low-dose exposure protocols to better mimic real-world conditions. Third, PM_2.5_ samples used in this study were collected from a single location and time period, and their physicochemical composition (metals, PAHs, and endotoxin) may not be representative of PM_2.5_ from all sources. PM_2.5_ composition varies considerably across regions, seasons, and pollution sources (traffic, industry, and dust), potentially leading to distinct biological effects. Consequently, generalizing our findings to other types of PM_2.5_ should be done with caution. Future studies should collect PM_2.5_ samples from diverse sources to compare their capacity to induce ferroptosis and identify the key toxic components responsible for exacerbating asthma. Fourth, regarding translational challenges, macrophage-specific LCN2 knockdown in this study was achieved using an AAV9 vector. Although this strategy provides valuable proof-of-concept evidence, it remains at a preclinical stage, and a considerable gap exists before clinical application. The safety, immunogenicity, delivery efficiency, and long-term effects of AAV vectors require further evaluation. Consequently, given the above limitations, future research should focus on identifying the key components of PM_2.5_ that induce ferroptosis in airway macrophages, validating our findings across multiple asthma models and with PM_2.5_ samples from different sources, and exploring the clinical translational potential of LCN2 as a therapeutic target for asthma.

## 5 Conclusion

In summary, our findings confirmed that PM_2.5_ exposure activates the TF Fra2, which upregulates LCN2 transcription and expression. This cascade inhibits mitophagy, promotes iron accumulation, and induces ferroptosis in M2 macrophages, ultimately aggravating asthma pathology by disrupting M1/M2 macrophage homeostasis.

## Funding

This work was supported by the Fundamental Research Program of Shanxi Province (202403021212216), the Science Foundation of Shanxi Bethune Hospital (2023RC53), and the Research and Innovation Team Project for Scientific Breakthroughs at Shanxi Bethune Hospital (2024AOXIANG01 and 2024ZHANCHI09).

## CRediT authorship contribution statement

**Caihong Wang:** Writing – original draft, Writing – review & editing. **Shutong Yang:** Data curation, Validation. **Zhihong Zhang:** Methodology. **Hongli Gao:** Investigation. **Qian Niu:** Methodology. **Jing Wu:** Investigation. **Sanai Lv:** Data curation. **Qi Mei:** Methodology. **Chunyan Gao:** Data curation. **Yukai Jing:** Supervision. **Xiansheng Liu:** Funding acquisition, Project administration.

## Declaration of competing interest

The authors declare that they have no known competing financial interests or personal relationships that could have appeared to influence the work reported in this paper.

## Data Availability

Data will be made available on request.
