# Supplementary material for: Targeting the Fra2/LCN2 axis attenuates PM2.5-aggravated asthma by suppressing M2 macrophage ferroptosis
Source: Redox Biol. 2026 May 26;94:104235. doi: 10.1016/j.redox.2026.104235 (PMC13241744; doi:10.1016/j.redox.2026.104235)
Supplement: Multimedia component 1 [file mmc1.docx]

**Targeting the Fra2/LCN2 Axis Attenuates PM_2.5_-aggravated Asthma by Suppressing M2 Macrophage Ferroptosis**

**Materials and methods**

- 1. **Study subjects and clinical samples**

**1.1 Inclusion criteria for patients with asthma：**

1) The person was definitely diagnosed with asthma. 2) The age is randomly distributed between 18-64 years old. 3) They have no smoking history or have quit smoking for more than one year. 4) They have no other major diseases. 5) They signed the informed consent form.

**1.2 Exclusion criteria for patients with asthma：**

1) The person with other allergic diseases. 2) In addition to asthma, there are other respiratory diseases that seriously affect the pulmonary function, such as tuberculosis, chronic obstructive pulmonary disease, lung cancer, etc.

Adhering to the inclusion and exclusion criteria, 33 patients with asthma and blood plasma samples of all participants were collected. Their basic demographic characteristics are presented in Table S1. This investigation was approved by the Medical Ethics Committee of Shanxi Medical University (No. 2018LL233), and all participants have signed the informed consent.

- 1. **Assessment of PM_2.5_ exposure concentration**

PM_2.5_ concentration distribution data were from Tracking Air Pollution in China. PM_2.5_ concentrations experienced by the participants were recorded during the 30 days prior to their involvement in the research. These data were based on the latitude and longitude of their residence locations and computed as mean exposure concentration (Xiao et al. 2022, Geng et al. 2021).

- 1. **Detection of plasma OVA-sIgE by ELISA**

The level of ovalbumin (OVA)-specific immunoglobulin E (OVA-sIgE) in mouse plasma was detected using a commercial enzyme-linked immunosorbent assay (ELISA) kit, with operations strictly following the manufacturer’s instructions (FineTest, Wuhan, China). The absorbance value of each well was measured at a wavelength of 450 nm using a microplate reader.

**Fig. S1.**

**
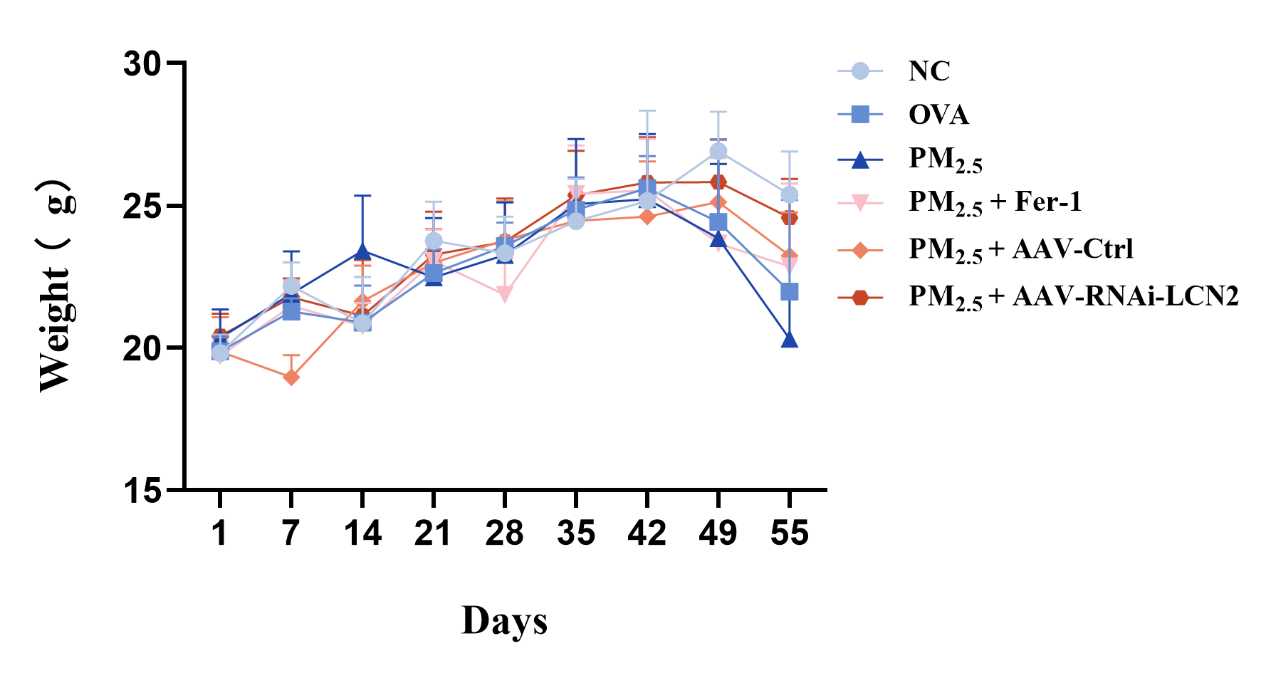
Body weight curve in a PM_2.5_-aggravated asthma mouse model.** Data are mean ± SD (n = 5). A repeated measures two‑way ANOVA was used to assess the effects of group, time, and their interaction. Significant effects were found for time (*P* < 0.0001), group (*P* = 0.0002), and group × time interaction (*P* < 0.0001).

**Fig. S2.**

**
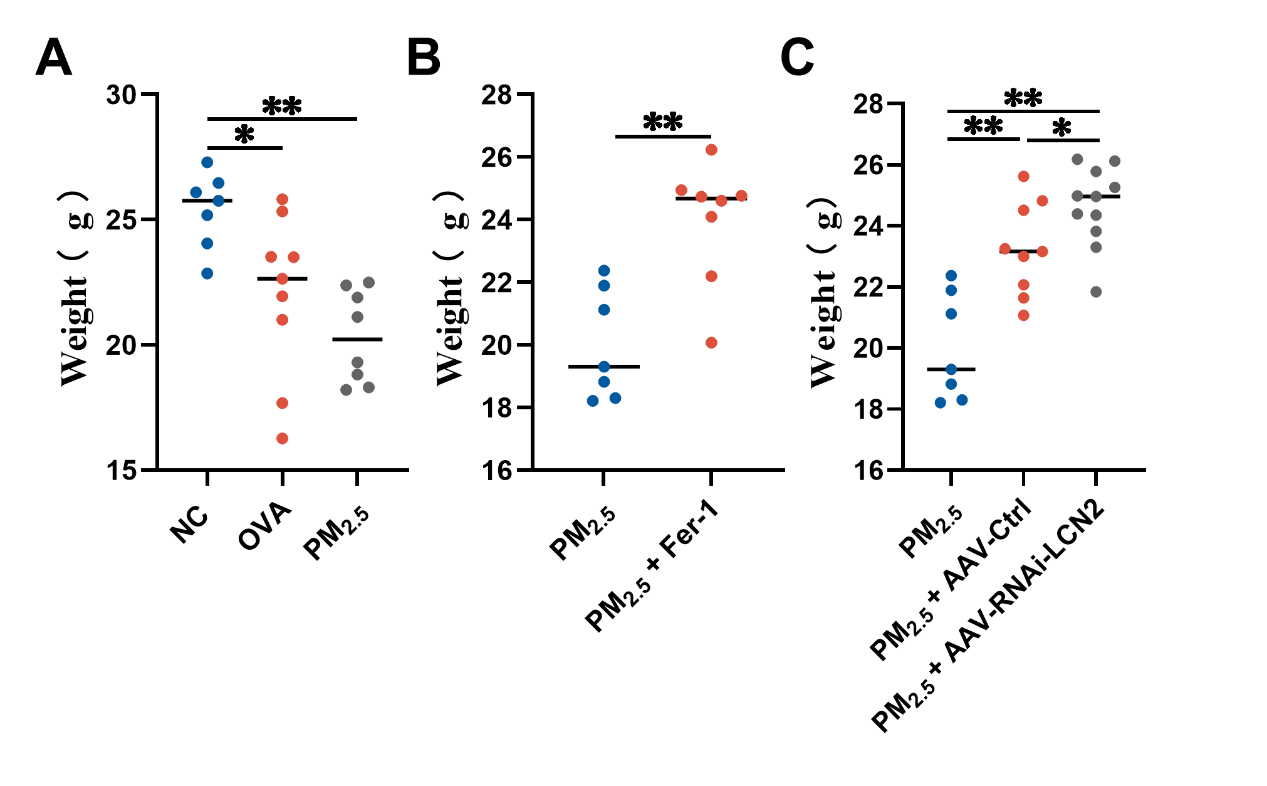
Body weight in different mouse groups. (A)** PM_2.5_-aggravated asthma mouse model. **(B)** Fer-1 treatment in a PM_2.5_-aggravated murine asthma model. **(C)** PM_2.5_-aggravated asthma mouse model following AAV9-mediated macrophage-specific LCN2 knockdown. (n = 6-11). Values are expressed as the means ± SD. * indicates *P* < 0.05, ** indicates *P* < 0.01and ns for no significance.

**Fig. S3.**

**
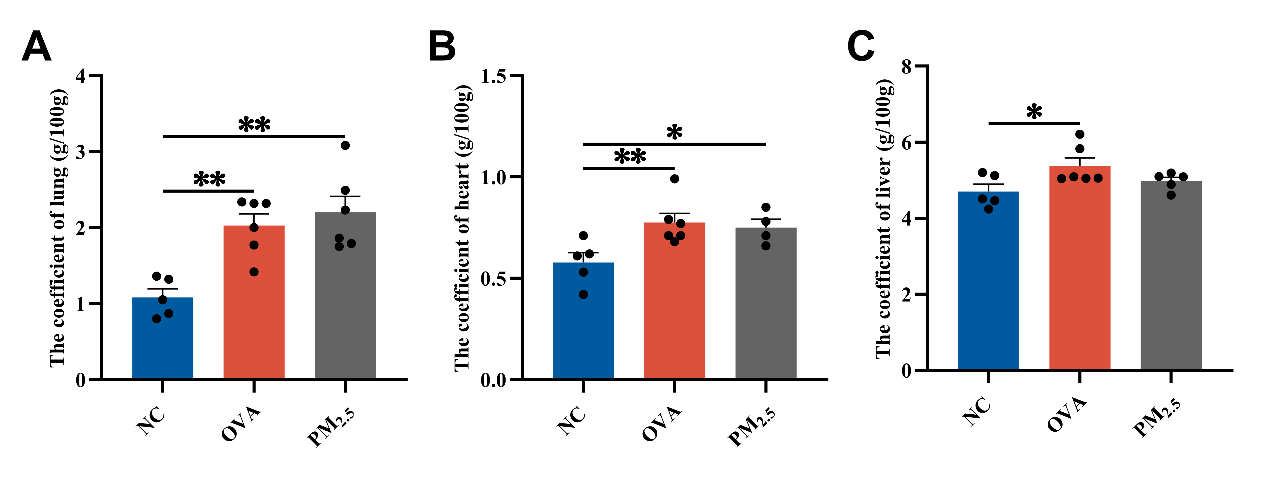
Organ coefficients in a PM_2.5_-aggravated murine asthma model.** **(A)** Lung. **(B)** Heart. **(C)** Liver. (n = 6). Values are expressed as the means ± SD. * indicates *P* < 0.05, ** indicates *P* < 0.01and ns for no significance.

**Fig. S4.**

**
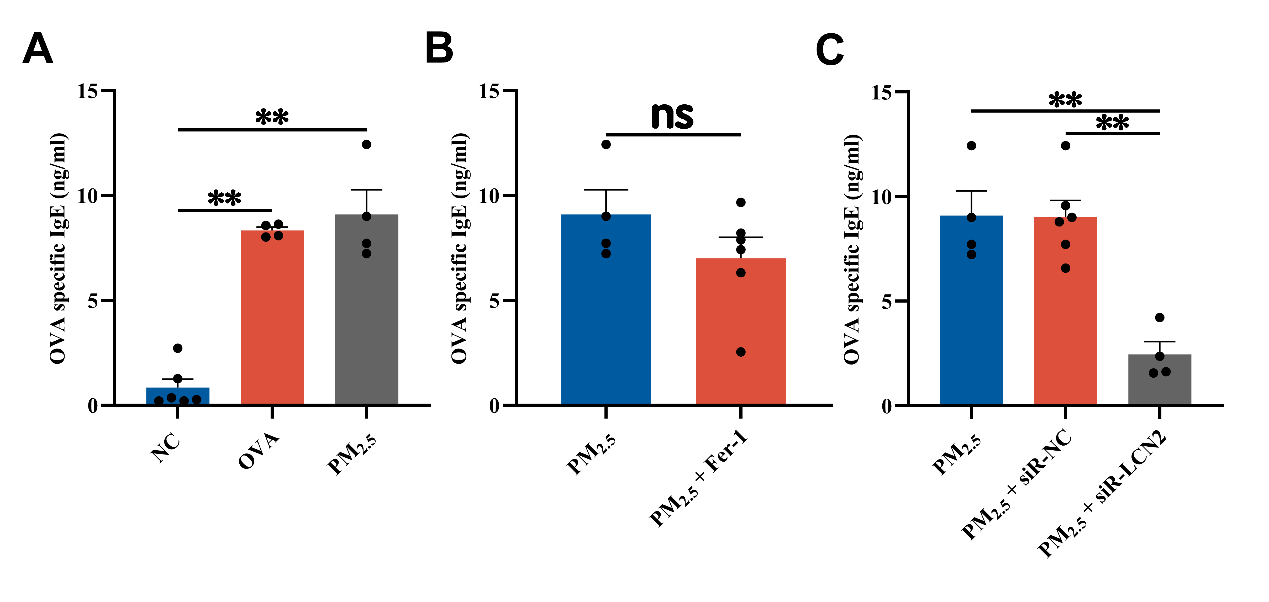
**

**The level of OVA- specific IgE (OVA-sIgE) in mouse plasma. (A)** Concentrations of OVA-sIgE in plasma of PM_2.5_-aggravated asthma mouse model. **(B)** Concentrations of OVA-sIgE in plasma following Fer-1 treatment in a PM_2.5_-aggravated murine asthma model. **(C)** Concentrations of OVA-sIgE in plasma of a PM_2.5_-aggravated asthma mouse model following AAV9-mediated macrophage-specific LCN2 knockdown. (n = 6). Values are expressed as the means ± SD. * indicates *P* < 0.05, ** indicates *P* < 0.01and ns for no significance.

**Fig. S5.**


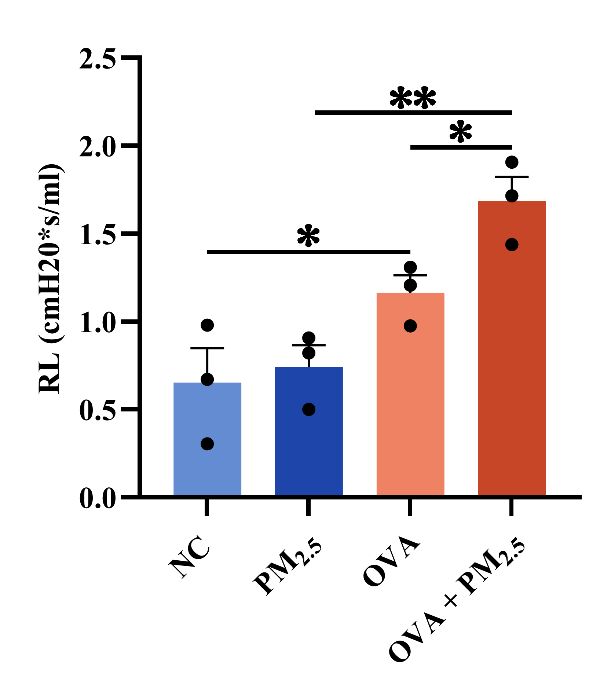


**The effect of PM_2.5_ on airway resistance (RL) in asthmatic mice.** (n = 3). Values are expressed as the means ± SD. * indicates *P* < 0.05, ** indicates *P* < 0.01.

**Fig. S6.**

**
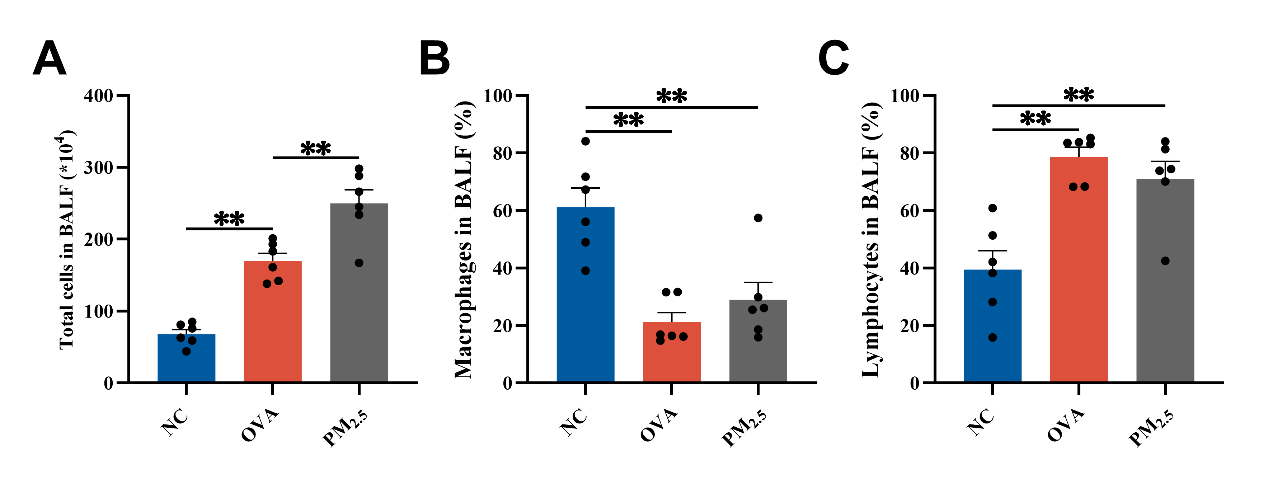
** **Effects of PM_2.5_ on total cell count and differential leukocyte count in BALF of asthmatic mice. (A)** Total cells. **(B)** Percentage of macrophages. **(C)** Percentage of lymphocytes. (n = 6). Values are expressed as the means ± SD. * indicates *P* < 0.05, ** indicates *P* < 0.01and ns for no significance.

**Fig. S7.**


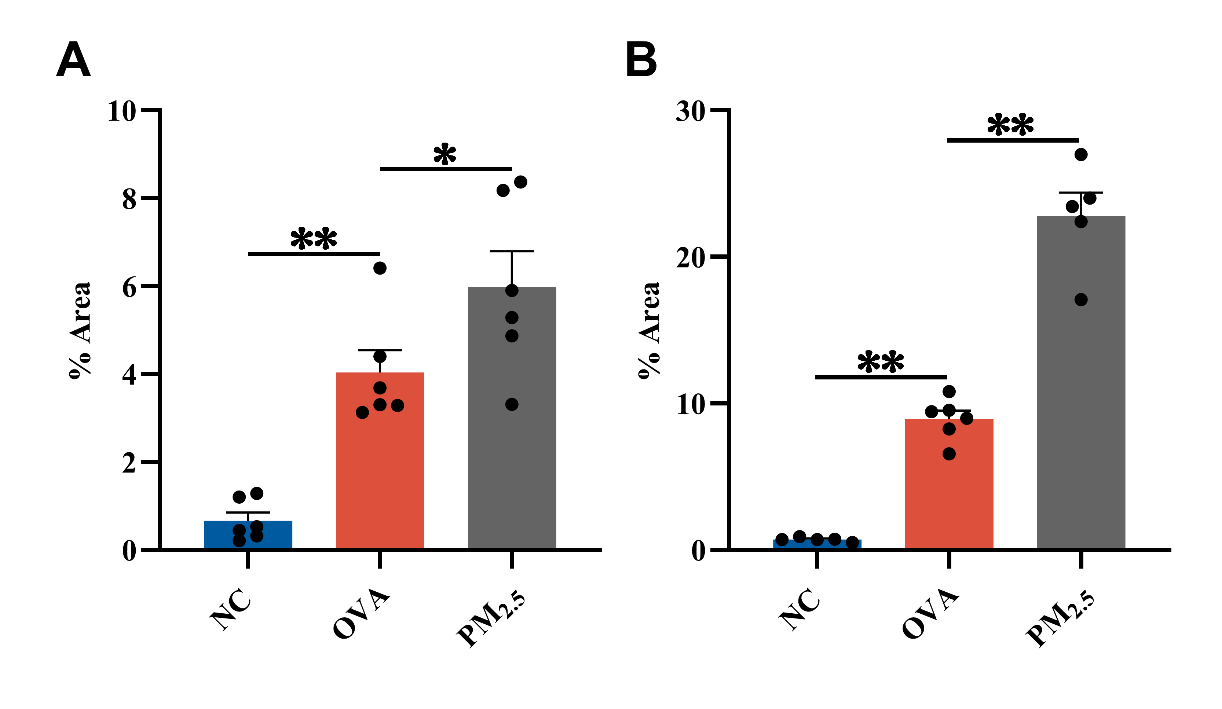


**Effects of PM_2.5_ exposure on lung histopathology and mucus secretion in asthmatic mice. (A)** HE. **(B)** PAS. (n = 6). Values are expressed as the means ± SD. * indicates *P* < 0.05, ** indicates *P* < 0.01.

**Fig. S8.**


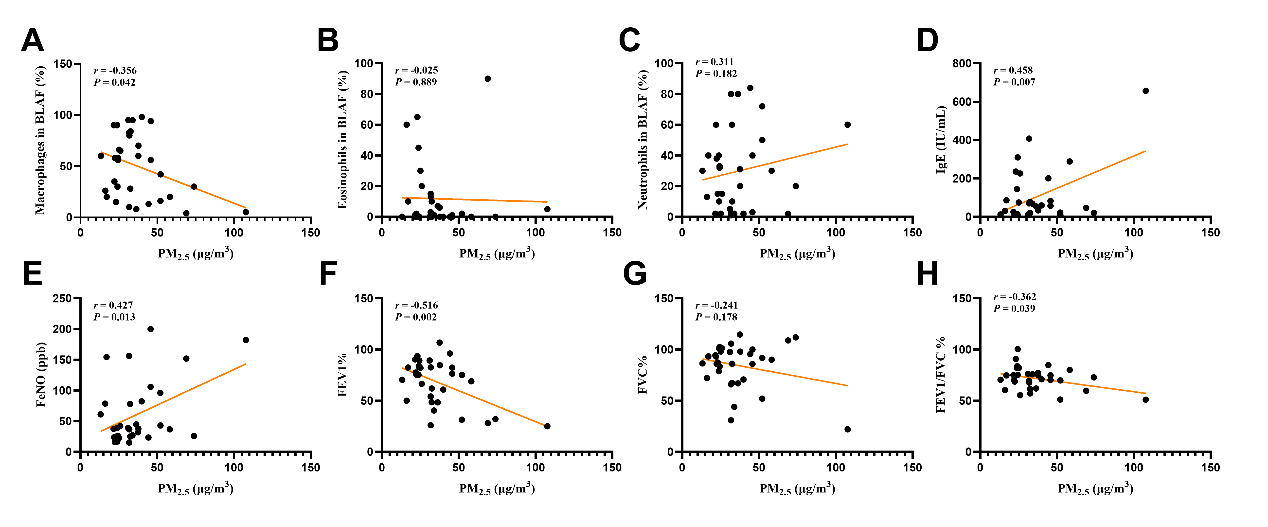


**The correlation between PM_2.5_ exposure concentration and pulmonary function in patients with asthma. (A)** The proportion of macrophages in BALF. **(B)** The proportion of eosinophils in BALF. **(C)** The proportion of neutrophils in BALF. **(D)** IgE. **(E)** FeNO. **(F)** FEV1 as a percentage of the predicted normal value (FEV1%). **(G)** Forced Vital Capacity as a percentage of the predicted normal value (FVC%). **(H)** FEV1/FVC%. (n = 33). Values are expressed as the means ± SD. * indicates *P* < 0.05, ** indicates *P* < 0.01and ns for no significance.

**Fig. S9.**


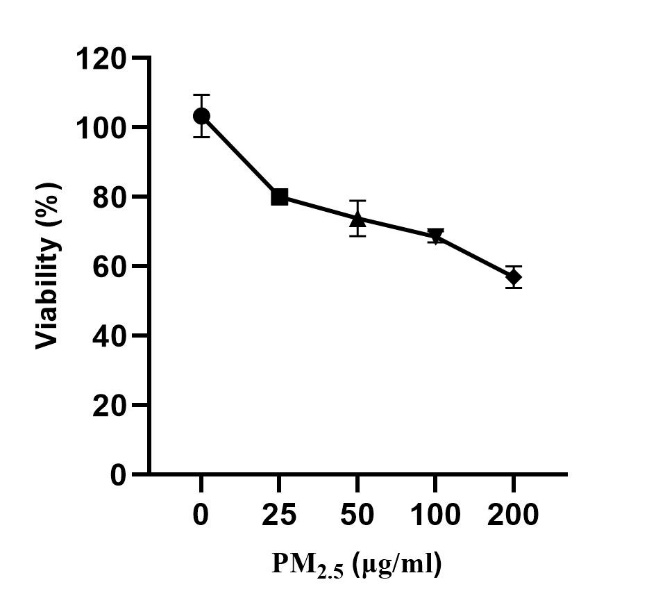


**Effects of PM_2.5_ at various concentrations on cell viability of RAW264.7 cells measured by CCK-8 assay.** Data are mean ± SD (n = 5). One‑way ANOVA followed by Tukey’s post‑hoc test was performed. ** *P* < 0.001 vs control group (0 μg/mL) for all tested concentrations.

**Fig. S10.**


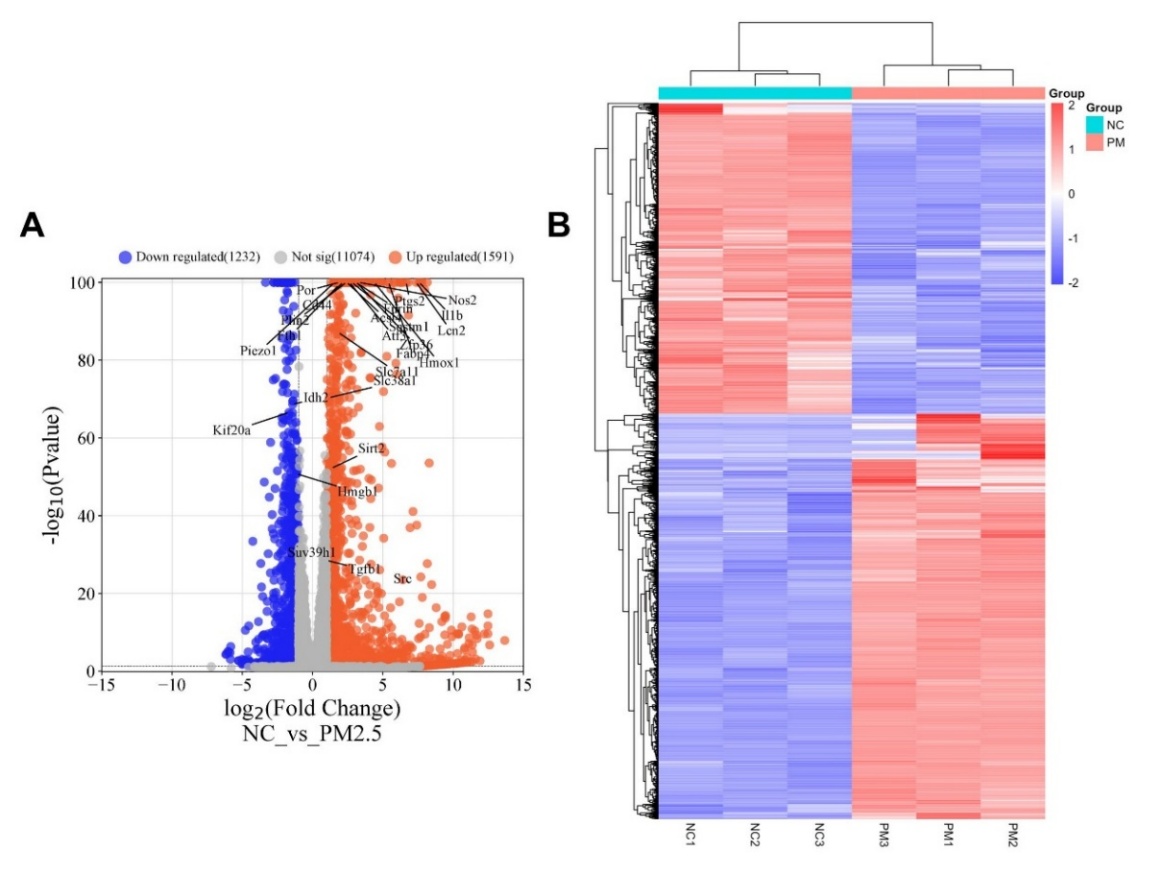
 **Differentially expressed genes (DEGs) in the transcriptome of RAW264.7 cells after PM_2.5_ stimulation. (A)** Volcano plot. **(B)** Cluster heatmap.

**Fig. S11.**
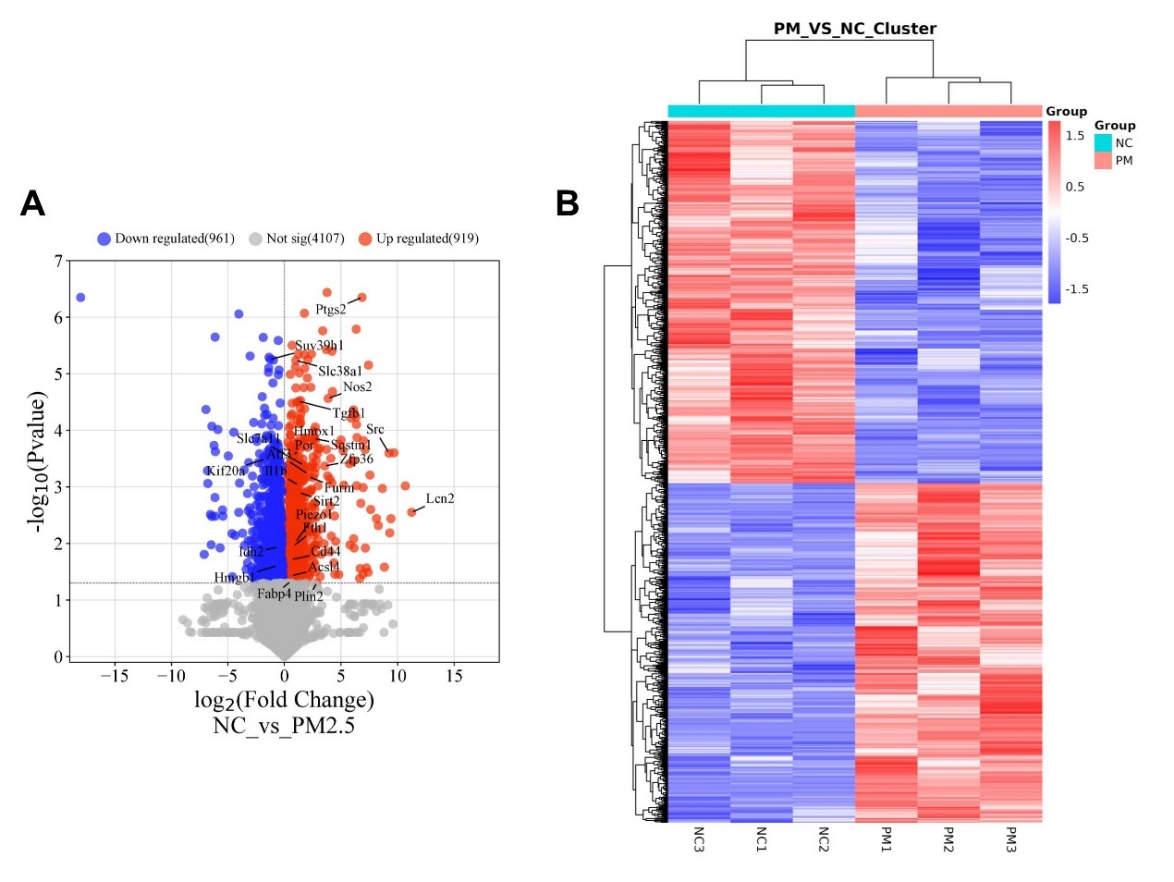
 **Proteomic analysis of RAW264.7 cells following PM_2.5_ stimulation.** **(A)** Volcano plot. **(B)** Cluster heatmap.

**Fig. S12.**

**
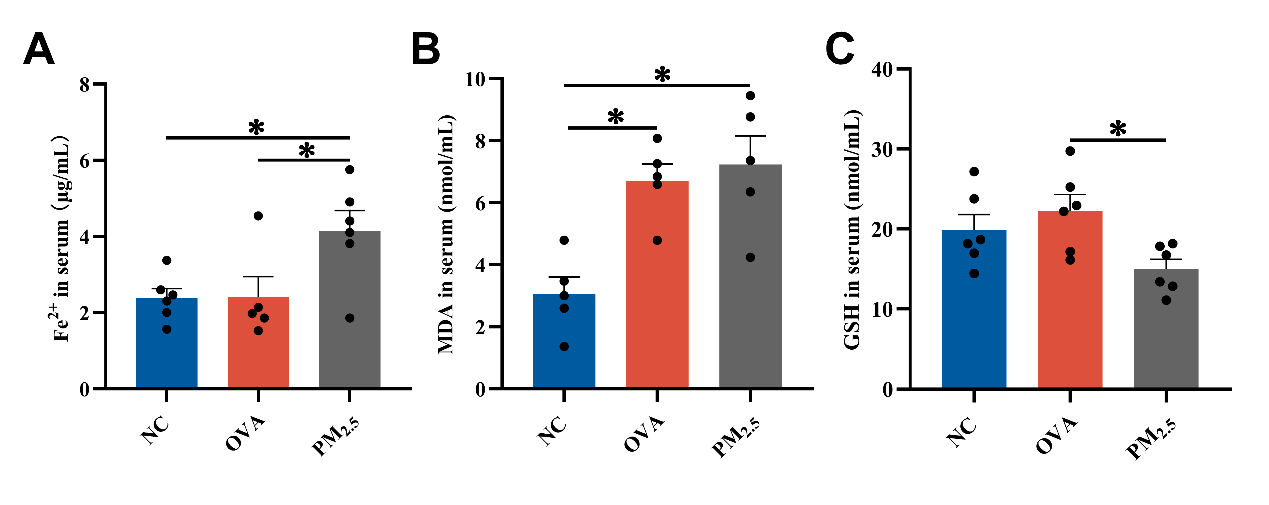
 Levels of Fe²⁺, MDA and GSH in plasma from PM_2.5_-aggravated asthmatic mice. (A)** Fe²⁺. **(B)** MDA. **(C)** GSH. (n = 6). Values are expressed as the means ± SD. * indicates *P* < 0.05, ** indicates *P* < 0.01and ns for no significance.

**Fig. S13.**


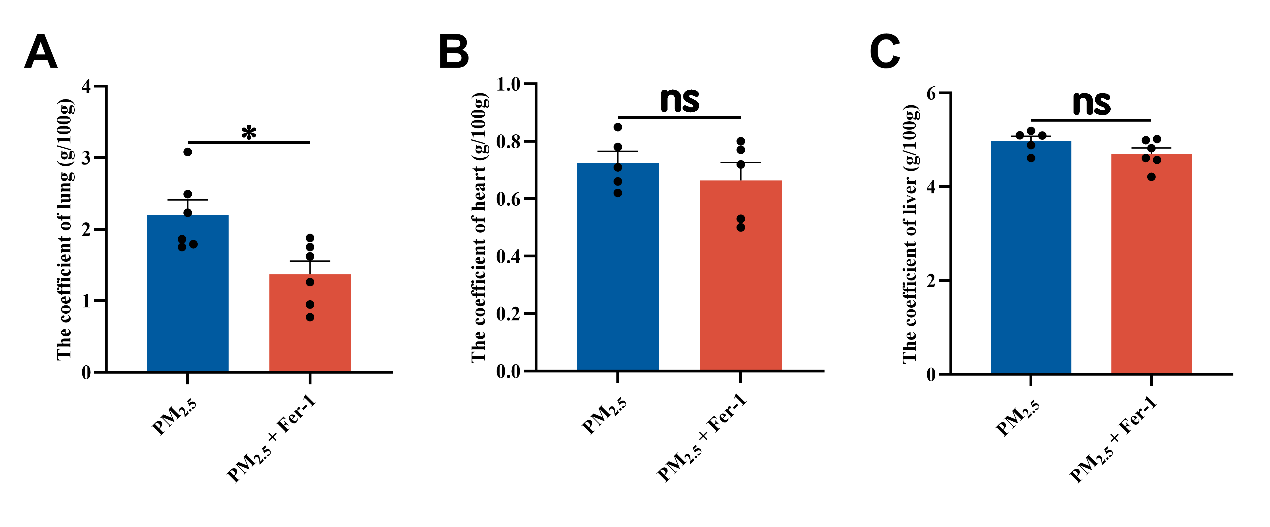


**Organ coefficients after Fer-1 treatment in a PM_2.5_-aggravated murine asthma model. (A)** Lung. **(B)** Heart. **(C)** Liver. (n = 6). Values are expressed as the means ± SD. * indicates *P* < 0.05, ** indicates *P* < 0.01and ns for no significance.

**Fig.S14**

**
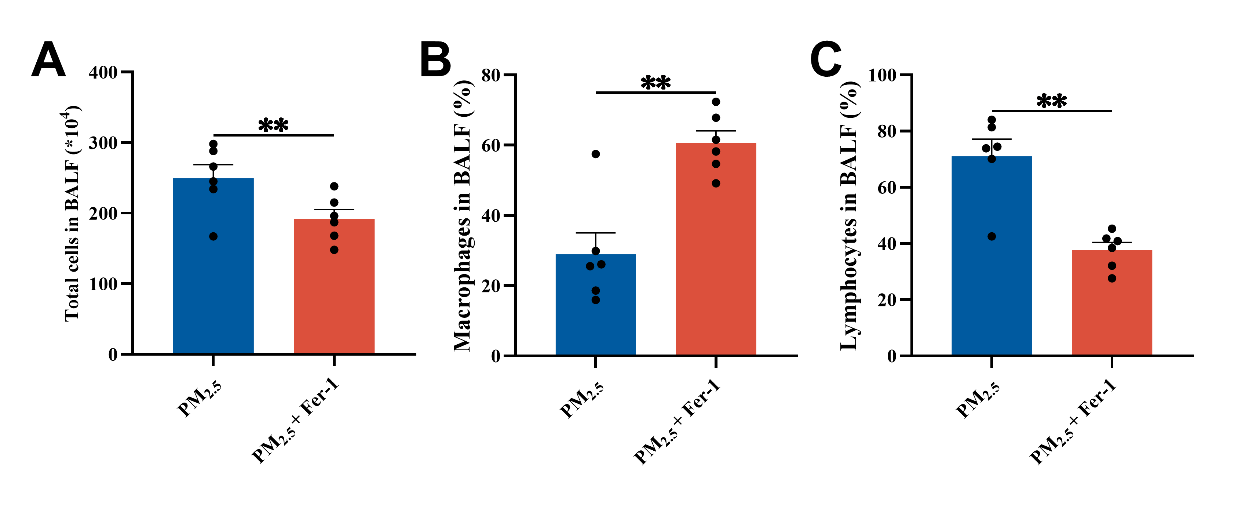
**

**Effects of Fer-1 on total cell count and differential leukocyte count in BALF of asthmatic mice. (A)** Total cells. **(B)** Percentage of macrophages. **(C)** Percentage of lymphocytes. (n = 6). Values are expressed as the means ± SD. * indicates *P* < 0.05, ** indicates *P* < 0.01.

**Fig. S15.**

**
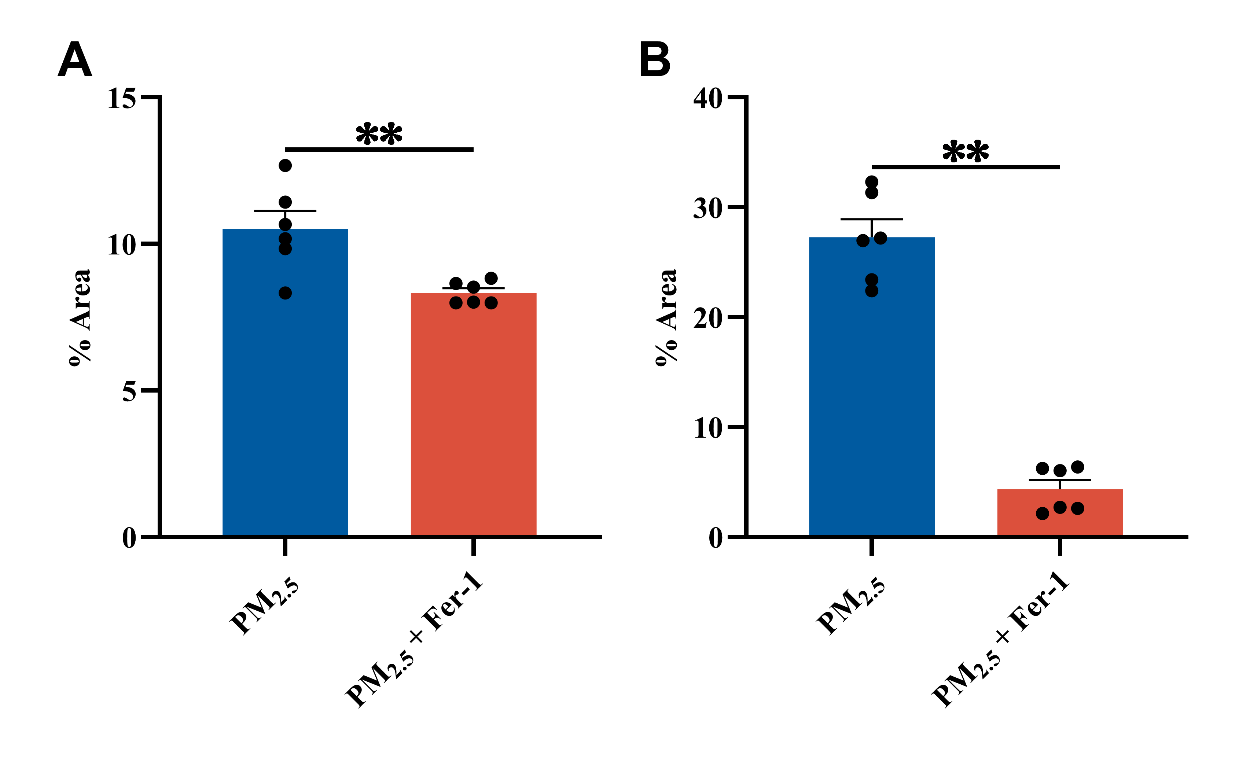
**

**Effects of Fer-1 on lung histopathology and mucus secretion in asthmatic mice. (A)** HE. **(B)** PAS. (n = 6). Values are expressed as the means ± SD. * indicates *P* < 0.05, ** indicates *P* < 0.01.

**Fig. S16.**

**
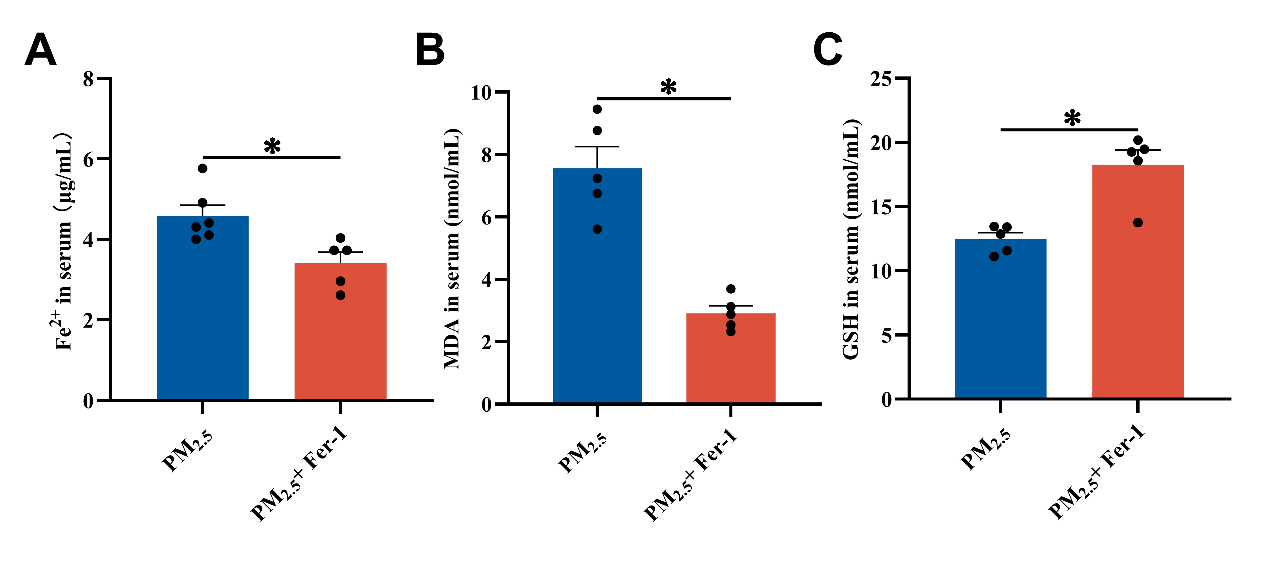
**

**Effects of Fer-1 on plasma levels of Fe²⁺, MDA, and GSH in a PM_2.5_-aggravated murine asthma model. (A)** Fe²⁺. **(B)** MDA. **(C)** GSH. (n = 6). Values are expressed as the means ± SD. * indicates *P* < 0.05, ** indicates *P* < 0.01and ns for no significance.

**Fig. S17.**


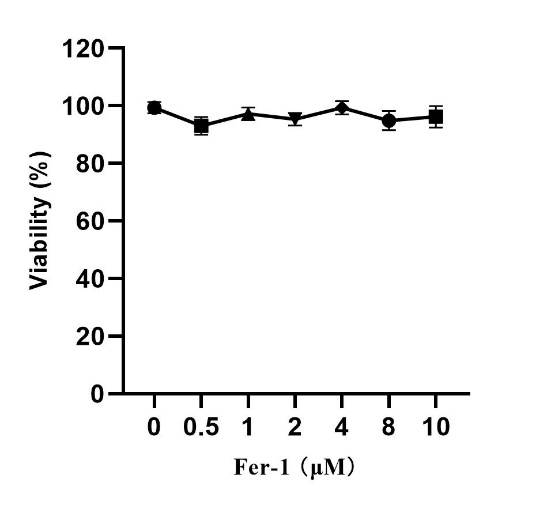


**Effects of Fer-1 at various concentrations on cell viability of RAW264.7 cells measured by CCK-8 assay.** Data are mean ± SD (n = 5). One‑way ANOVA revealed no significant difference among groups (*P* > 0.05), indicating no cytotoxicity within the tested concentration range (0–10 μM).

**Fig. S18.**

**
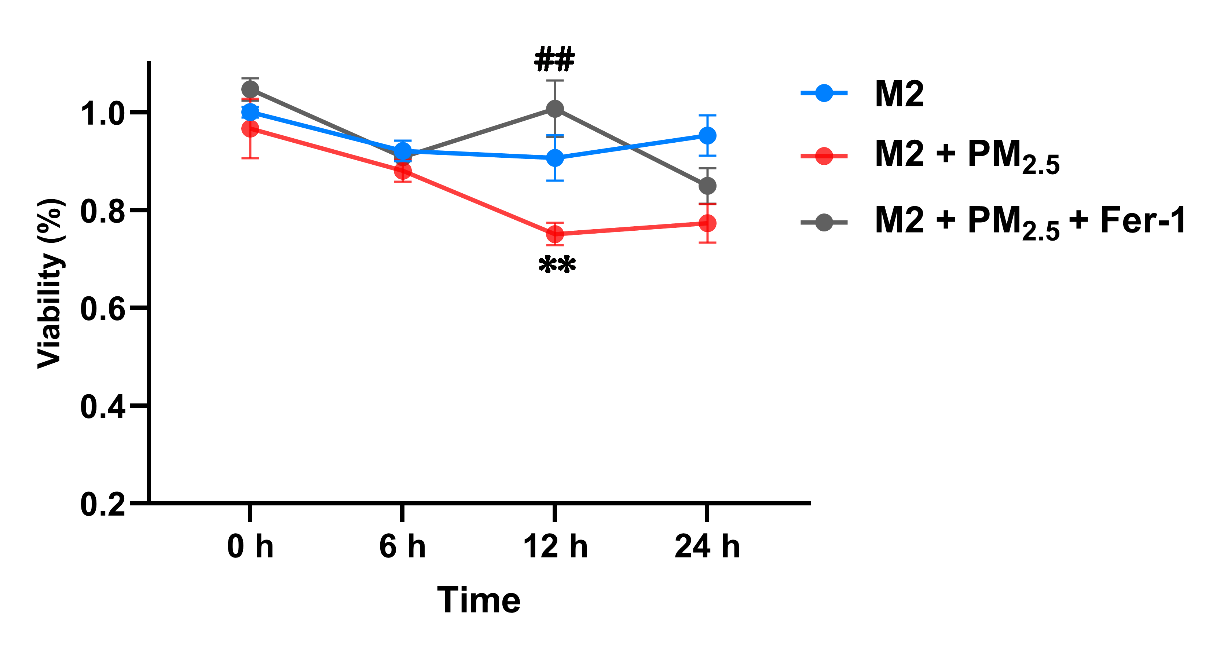
**

**Cell viability of RAW264.7 cells treated with PM_2.5_ and Fer-1 at various time points, assessed by the CCK-8 assay.** **Data are mean ± SD (n = 5). Two‑way ANOVA was used to assess treatment, time, and their interaction. Significance indicators:**

*****P* < 0.01 vs M2 group (PM_2.5_ at 12 h); ## *P* < 0.01 vs M2 + PM_2.5_ group (M2 + PM_2.5_ + Fer‑1 at 12 h). Two‑way ANOVA revealed significant effects of treatment (*P* = 0.0005), time (*P* = 0.0002), and their interaction (*P* = 0.0386).**

**Fig. S19.**

**
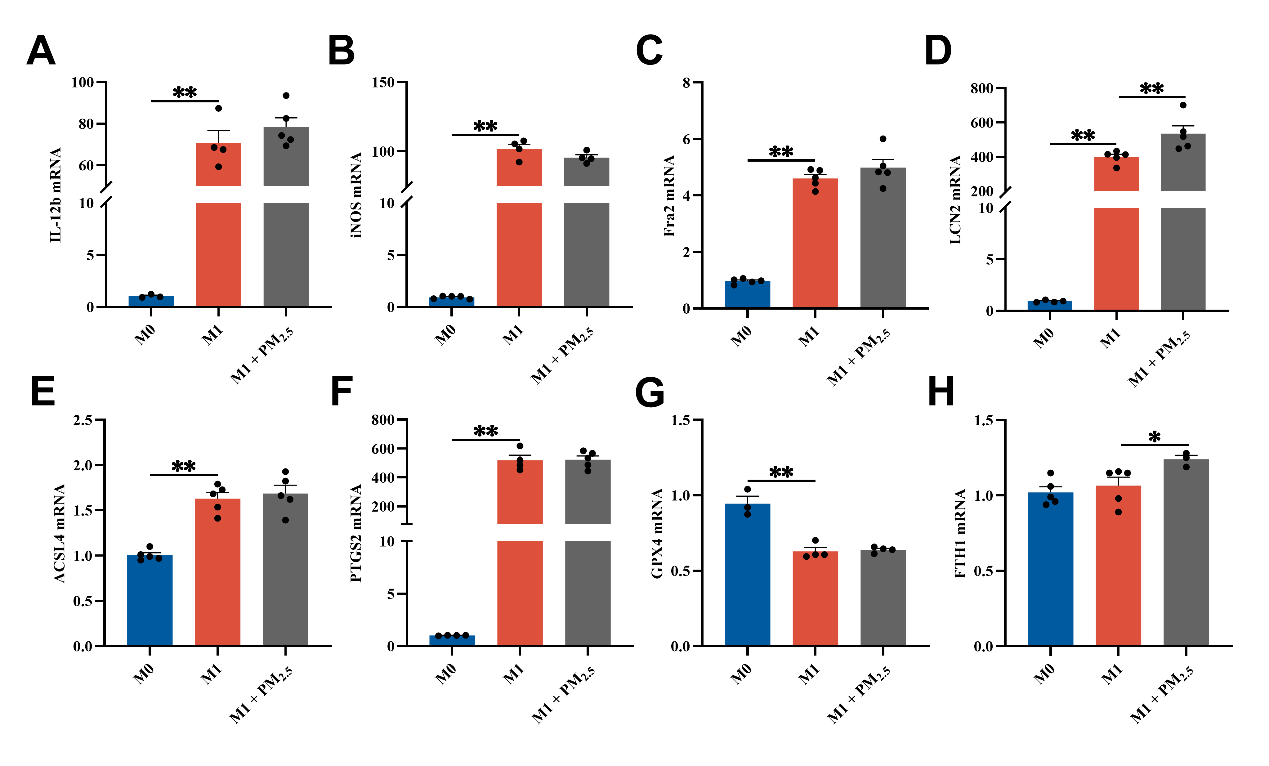
**

**Effects of PM_2.5_ on mRNA expression levels of ferroptosis-related genes in M1 macrophages. (A)** IL-12b. **(B)** iNOS. **(C)** Fra2. **(D)** LCN2. **(E)** ACSL4. **(F)** PTGS2. **(G)** GPX4. **(H)** FTH1. (n = 5). Values are expressed as the means ± SD. * indicates *P* < 0.05, ** indicates *P* < 0.01and ns for no significance.

**Fig. S20.**

**
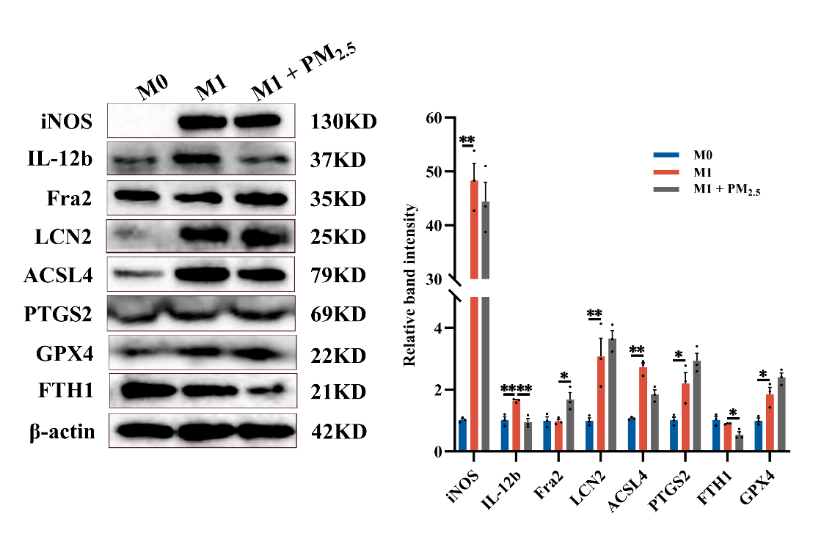
**

**Effects of PM_2.5_ on protein expression levels of ferroptosis-related proteins in M1 macrophages. (A)** IL-12b. **(B)** iNOS. **(C)** Fra2. **(D)** LCN2. **(E)** ACSL4. **(F)** PTGS2. **(G)** GPX4. **(H)** FTH1. (n = 3). Values are expressed as the means ± SD. * indicates *P* < 0.05, ** indicates *P* < 0.01and ns for no significance.

**Fig. S21.**


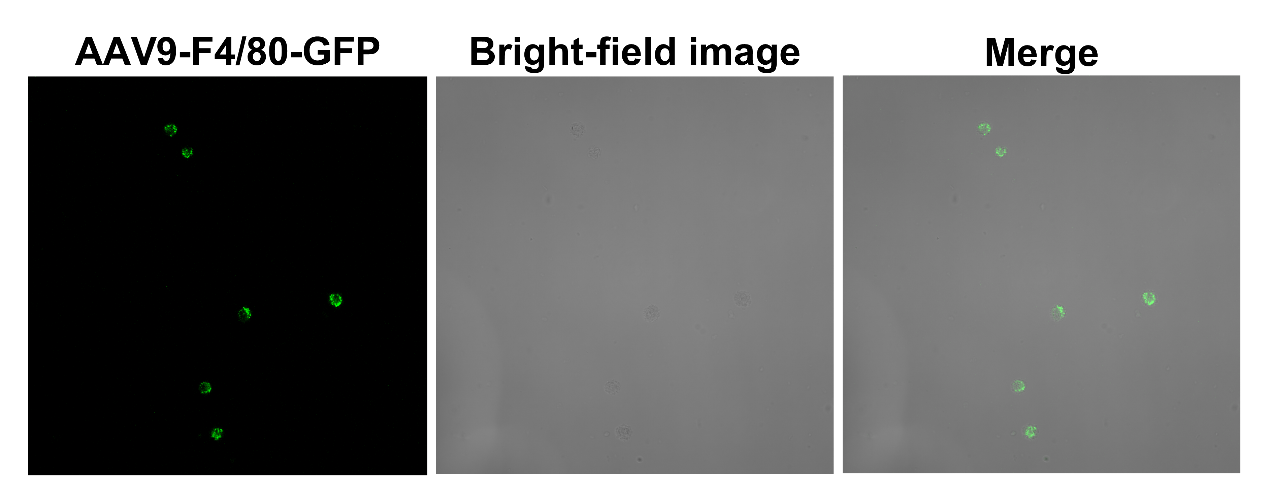
 **Representative fluorescence images of macrophages in the BALF of mice after intratracheal instillation of AAV9-F4/80-GFP.**

**Fig. S22.**

**
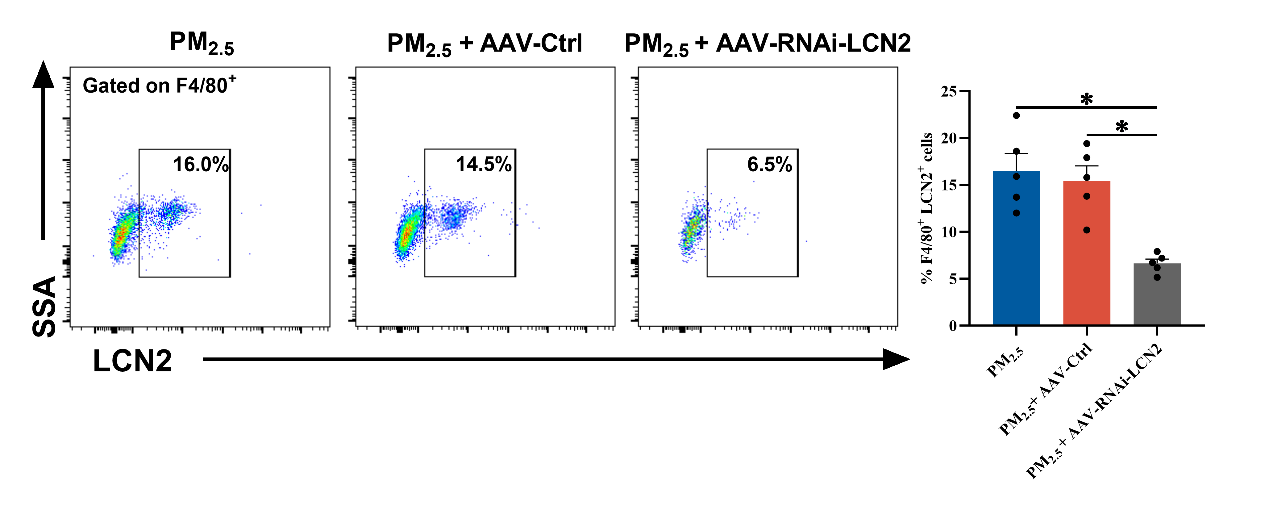
**

**The proportion of F4/80⁺LCN2⁺ cells in the BALF of mice after macrophage-specific knockdown of LCN2 mediated by the AAV9 vector.** (n = 5). Values are expressed as the means ± SD. * indicates *P* < 0.05, ** indicates *P* < 0.01.

**Fig. S23.**

**
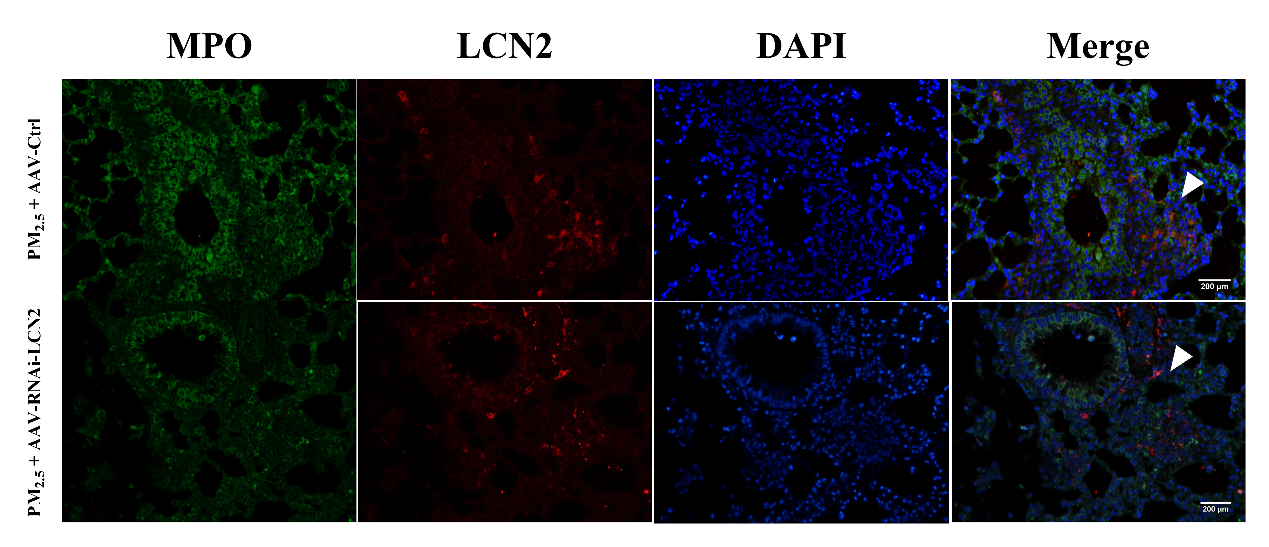
**

**The proportion of MPO⁺LCN2⁺ cells in the lung tissue of mice after macrophage-specific knockdown of LCN2 mediated by the AAV9 vector.** Scale bar: 200 μm.

**Fig. S24.**

**
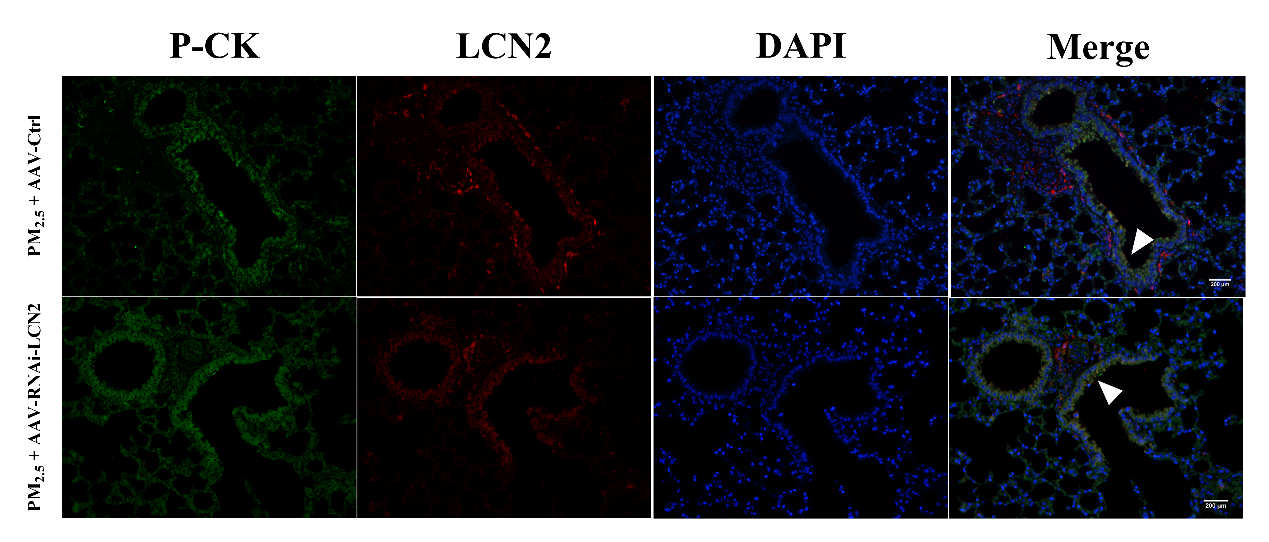
**

**The proportion of P-CK⁺LCN2⁺ cells in the lung tissue of mice after macrophage-specific knockdown of LCN2 mediated by the AAV9 vector.** Scale bar: 200 μm.

**Fig. S25.**

**
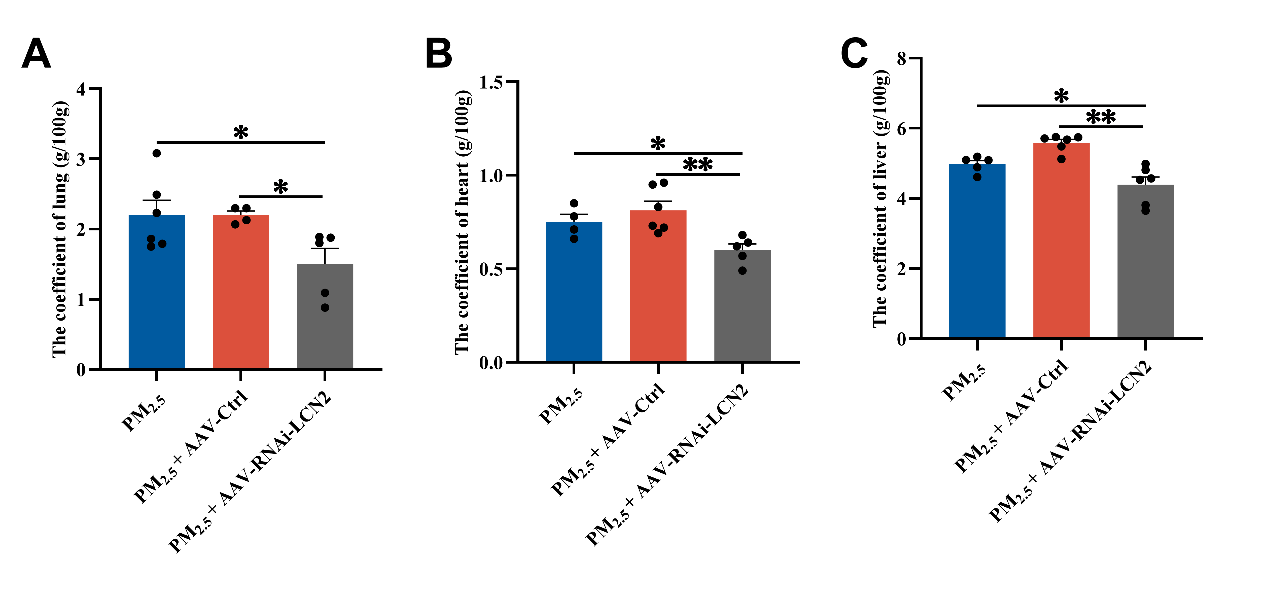
 Organ coefficients in PM_2.5_-aggravated asthma mouse model following AAV9-mediated macrophage-specific LCN2 knockdown. (A)** Lung. **(B)** Heart. **(C)** Liver. (n = 6). Values are expressed as the means ± SD. * indicates *P* < 0.05, ** indicates *P* < 0.01and ns for no significance.

**Fig. S26.**


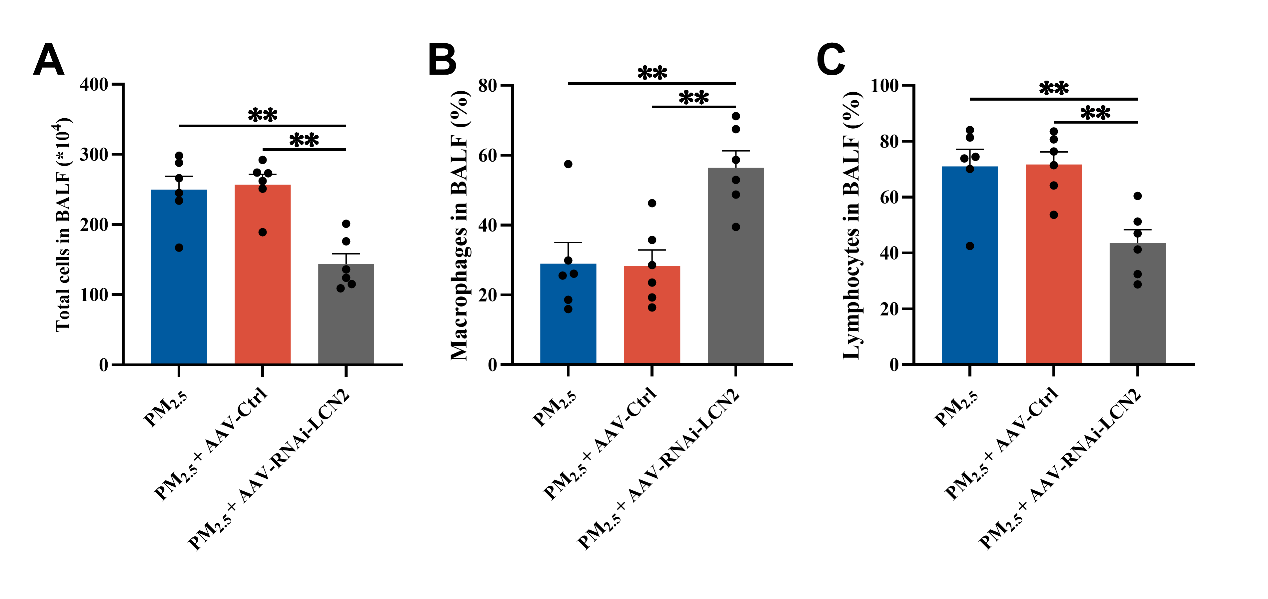


**Effects of AAV9-mediated macrophage-specific LCN2 knockdown on total and differential leukocyte counts in BALF. (A)** Total cells. **(B)** Percentage of macrophages. **(C)** Percentage of lymphocytes. (n = 6). Values are expressed as the means ± SD. * indicates *P* < 0.05, ** indicates *P* < 0.01.

**Fig. S27.**

**
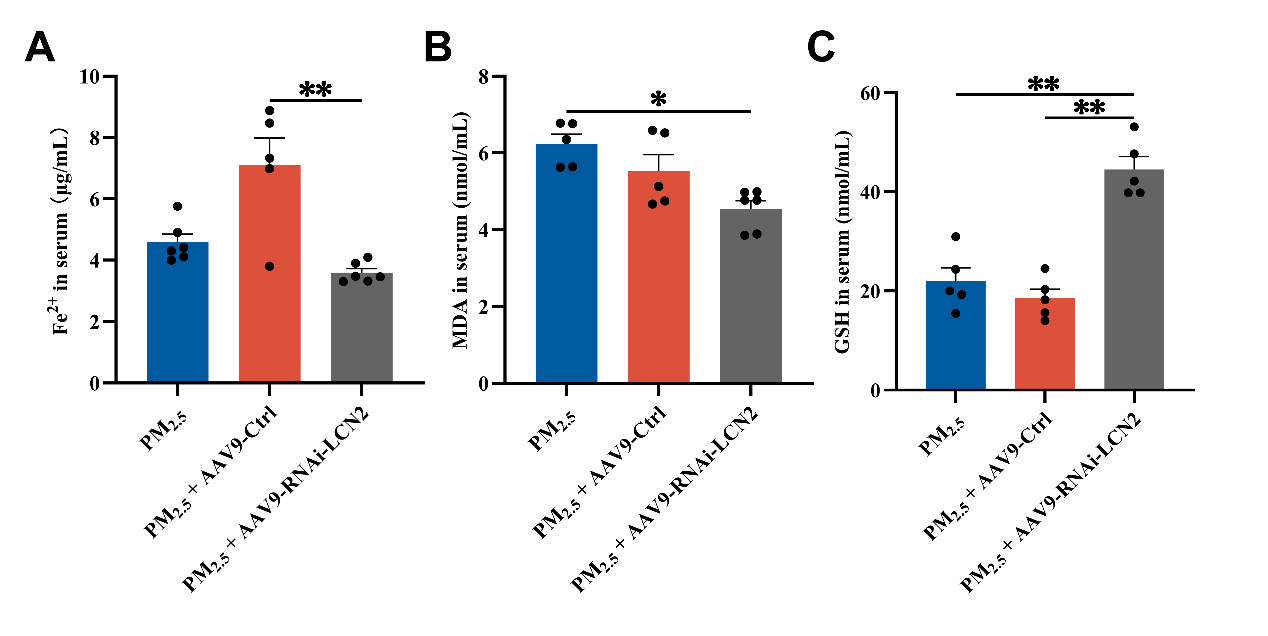
 Plasma levels of Fe²⁺, MDA, and GSH in a PM_2.5_-aggravated asthma mouse model following AAV9-mediated macrophage-specific LCN2 knockdown. (A)** Fe²⁺. **(B)** MDA. **(C)** GSH. (n = 6). Values are expressed as the means ± SD. * indicates *P* < 0.05, ** indicates *P* < 0.01and ns for no significance.

**Fig. S28.**


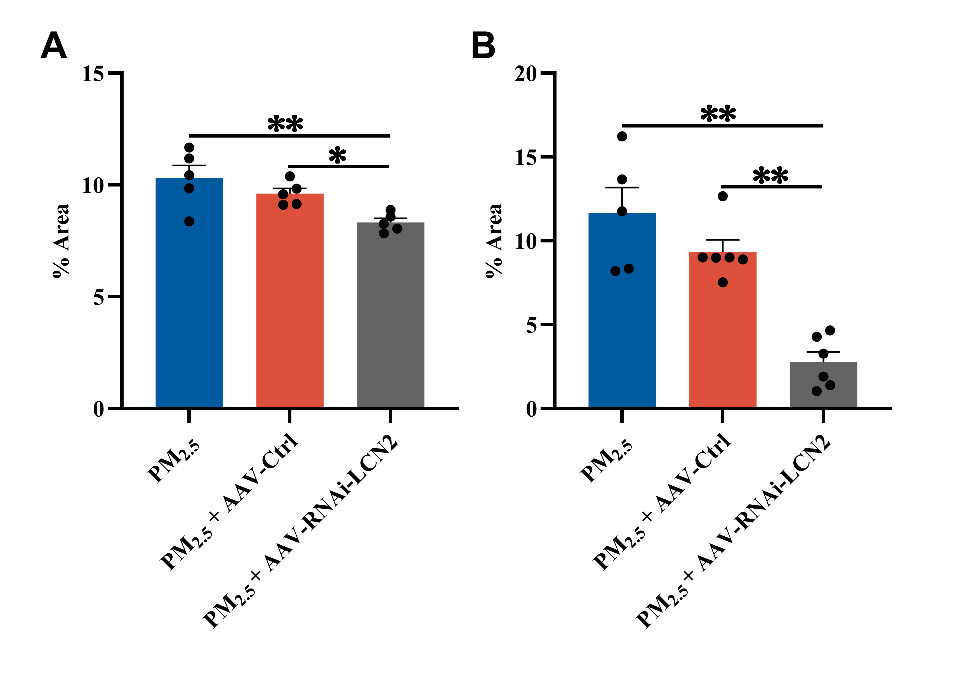


**Effects of macrophage-specific LCN2 knockdown on lung histopathology and mucus secretion in asthmatic mice. (A)** HE. **(B)** PAS. (n = 6). Values are expressed as the means ± SD. * indicates *P* < 0.05, ** indicates *P* < 0.01.

**Fig. S29.**

**
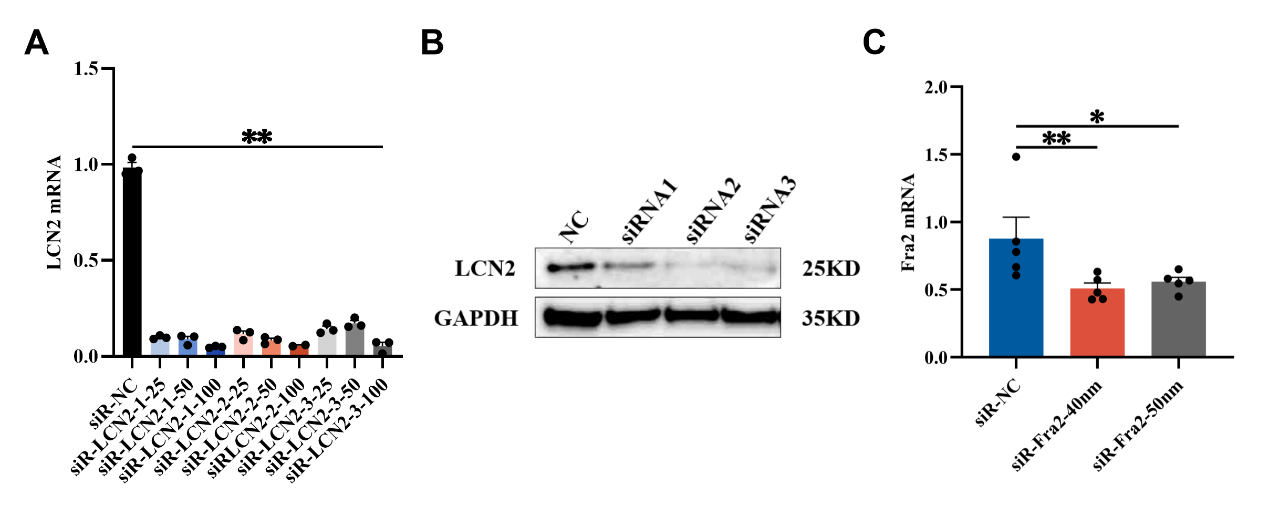
Screening of optimal sequences and concentrations for siR-Fra2 and siR-LCN2 transfection. (A)** LCN2 mRNA levels in RAW264.7 cells transfected with different siRNA sequences and concentrations as determined by RT-qPCR. **(B)** LCN2 protein levels in RAW264.7 cells transfected with different siRNA sequences and concentrations as determined by Western blot. **(C)** Fra2 mRNA levels in RAW264.7 cells transfected with different concentrations as determined by RT-qPCR. (n = 3-5). Values are expressed as the means ± SD. * indicates *P* < 0.05, ** indicates *P* < 0.01and ns for no significance.

**Fig. S30.**


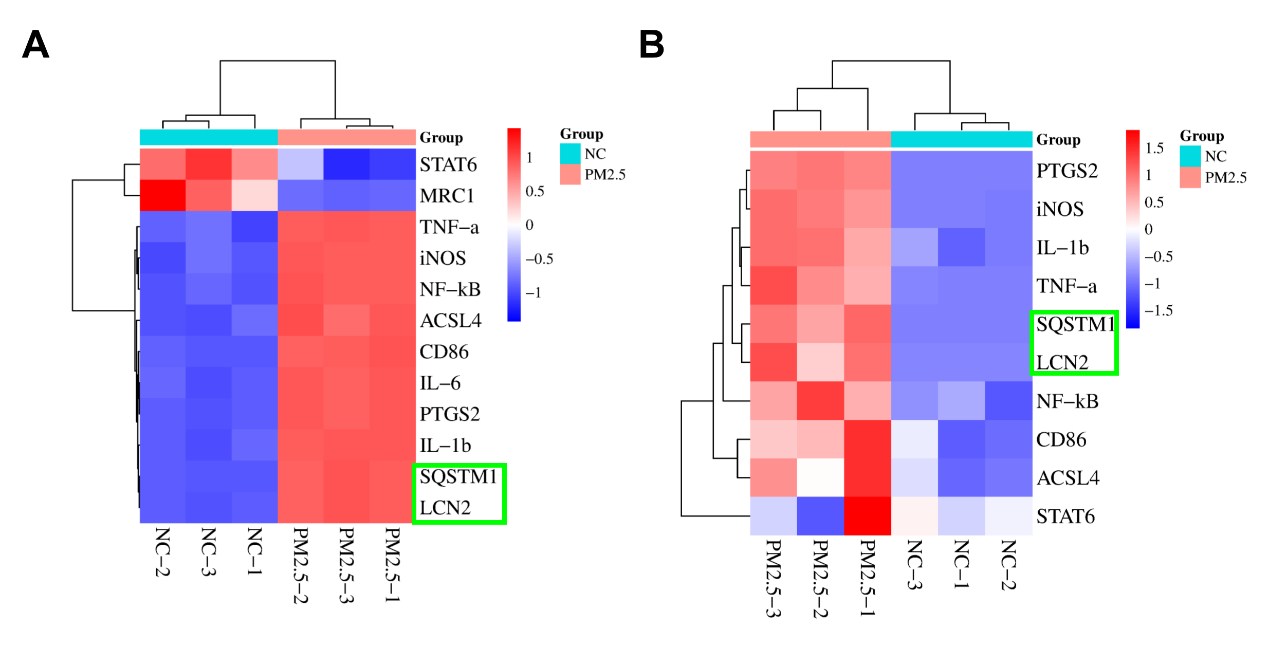
 **Clustered heatmap of differentially expressed genes associated with M1/M2 macrophages, ferroptosis, and mitophagy. (A)** Transcriptome. **(B)** Proteome.

**Fig. S31.**


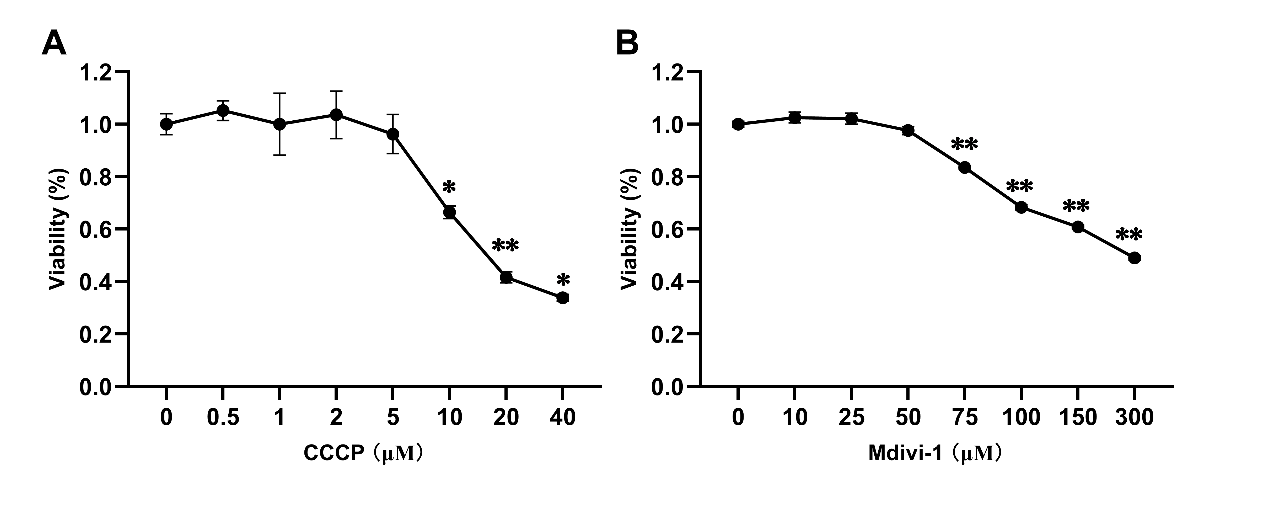


**Effects of CCCP and Mdivi-1 at various concentrations on cell viability of RAW264.7 cells measured by CCK-8 assay. (A)** CCCP. **(B)** Mdivi-1. Data are mean ± SD (n = 5). One‑way ANOVA followed by Tukey’s post‑hoc test was performed. * *P* < 0.05, ** *P* < 0.01 vs control group (0 μg/mL).

**Fig. S32.**

**
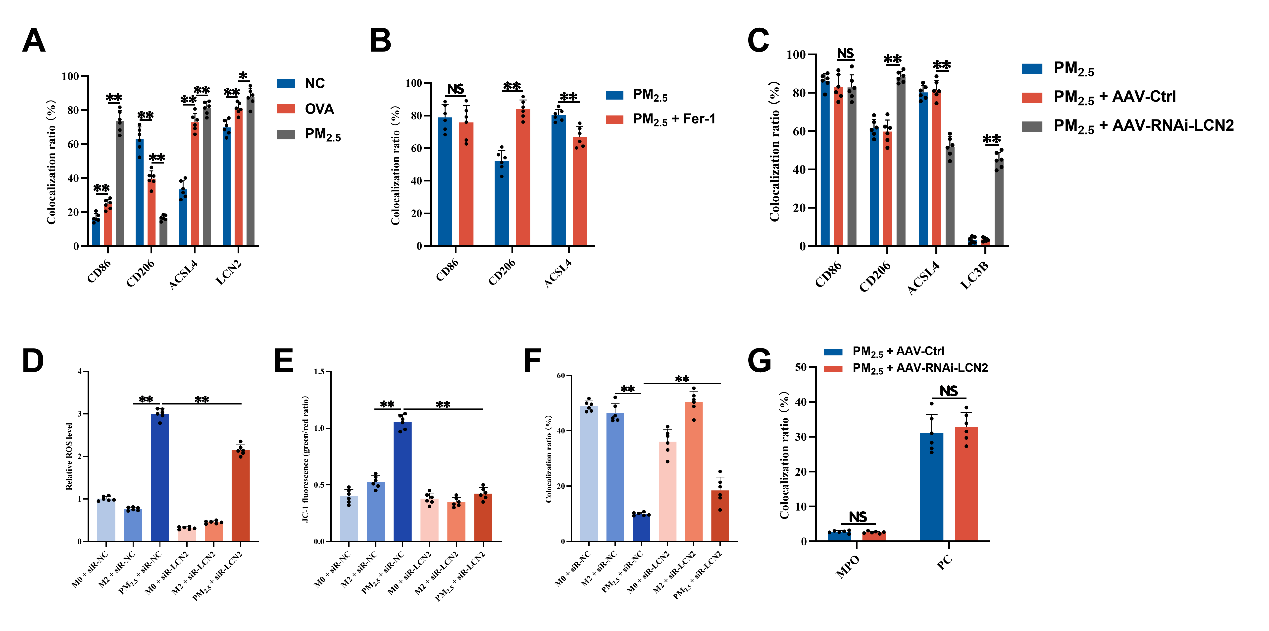
**

**Quantitative analysis of immunofluorescence staining results. (A)** Effect of PM_2.5_ on the immunofluorescence colocalization ratios of CD86/F4/80, CD206/F4/80, ACSL4/CD206, and LCN2/CD206 in lung tissue. **(B)** Effect of Fer-1 on the immunofluorescence colocalization ratios of CD86/F4/80, CD206/F4/80, ACSL4/CD206 in lung tissue. **(C)** Effect of AAV-mediated specific knockdown of LCN2 in macrophages on the immunofluorescence colocalization ratios of CD86/F4/80, CD206/F4/80, ACSL4/CD206, and LC3B/CD206 in lung tissue. **(D)** Quantitative fluorescence analysis of intracellular ROS levels detected by the DCFH-DA probe. **(E)** Quantitative analysis of the green/red fluorescence intensity ratio. **(F)** Knockdown of LCN2 in PM_2.5_-stimulated M2 macrophages resulted in significant changes in the immunofluorescence colocalization ratio of LC3B with mitochondria**. (G)** Effect of AAV-mediated specific knockdown of LCN2 in macrophages on LCN2 expression levels in neutrophils (MPO⁺) and epithelial cells (PC⁺) in lung tissue. (n = 6). Values are expressed as the means ± SD. * indicates *P* < 0.05, ** indicates *P* < 0.01and ns for no significance.

**Fig. S33.**

**
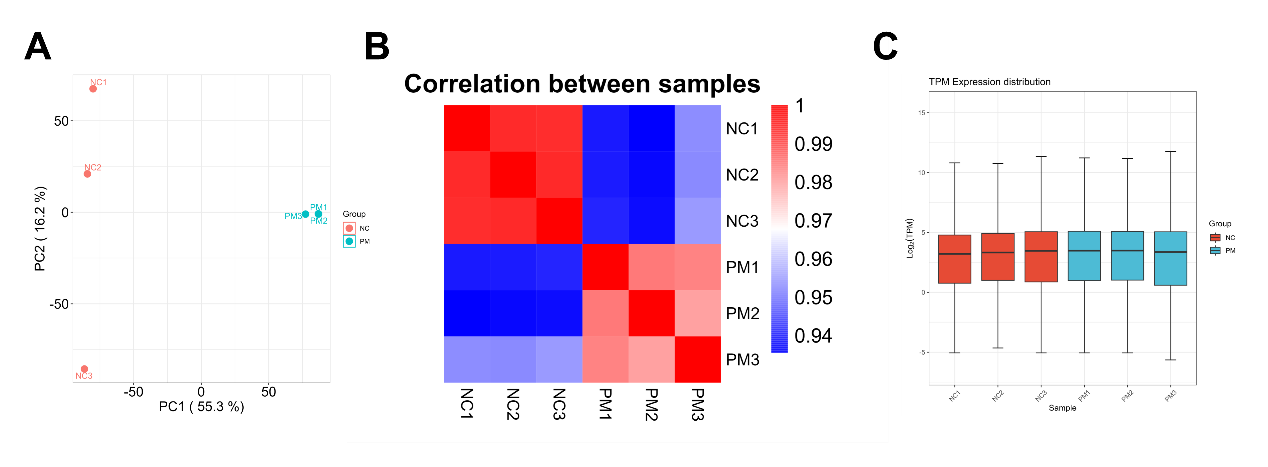
**

**Quality control and batch effect assessment of omics data. (A)** Principal component analysis (PCA) plot. **(B)** Sample correlation heatmap. **(C)** TPM boxplot.

**Tables S1.**

The baseline characteristics of asthmatic patients

| Basic characteristics | Asthma（n = 33） |
| --- | --- |
| Age (years)（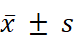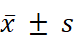） | 44.4 ± 16.30 |
| Sex [n (%)] |  |
| Male | 10（30.30） |
| Female | 23（69.70） |
| BMI（kg/m^2^）（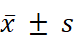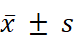） | 23.45 ± 6.80 |
| Education level [n (%)] |  |
| Junior High school and below | 8（24.20） |
| High school | 10（30.30） |
| College degree or above | 15（45.50） |
| Average monthly income per capita [n (%)] (%)] |  |
| < 3000元 | 18（54.50） |
| 3000–5000元 | 15（45.50） |
| VC (L) (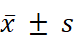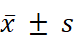) | 3.42 ± 0.99 |
| FVC (L) (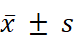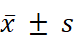) | 3.31 ± 1.11 |
| FEV1 (L) (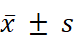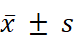) | 2.56 ± 0.94 |
| FEV1/FVC (%) (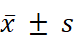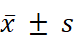) | 72.07 ± 10.92 |
| PM_2.5_ exposure (μg/m^3^) (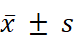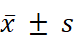) | 36.85 ± 20.51 |

BMI：Body Mass Index；VC：Vital Capacity；FVC：Forced Vital Capacity；FEV1：forced expiratory volume in 1s；FEV1/FVC：Ratio of FEV₁ to FVC.

**Table S2.**

Sequences of primers used in mice

| Gene name | Sequence (5'-3') |
| --- | --- |
| *Fra2* | F: GGAGGAGAAGCGTCGAATCC |
|  | R: GGGGCTGATTTTGCACACG |
| *LCN2* | F: TGGCCCTGAGTGTCATGTG |
|  | R: CTCTTGTAGCTCATAGATGGTGC |
| *ACSL4* | F: CTCACCATTATATTGCTGCCTGT |
|  | R: TCTCTTTGCCATAGCGTTTTTCT |
| *PTGS2* | F: GGGAGTCTGGAACATTGTGAA |
|  | R: GTGCACATTGTAAGTAGGTGGACT |
| *FTH1* | F: GCCGAGAAACTGATGAAGCTGC |
|  | R: GCACACTCCATTGCATTCAGCC |
| *CD206* | F: GTTCACCTGGAGTGATGGTTCTC |
|  | R: AGGACATGCCAGGGTCACCTTT |
| *Arg1* | F: GAAGAGTCAGTGTGGTGCTG |
|  | R: CCTTTCAGTTCCTTCAGGAGAA |
| *iNOS* | F: GGAGGTGACCATGGAGCAT |
|  | R: GCGCTGTGTGTCACAGAAGT |
| P62 | F: CCACCCCCTTTGTCTTGTAGT |
|  | R: GCCTGAAAAGGCATCACACAT |
| LC3 | F: GAGACATTCGGGACAGCAAT |
|  | R: CTATGTGGGTGCCTACGTTC |
| *β-actin* | Purchased from Biotechnology (B661302) |

**Table S3.**

Sequences of primers used in human

| Gene name | Sequence (5'-3') |
| --- | --- |
| *Fra2* | F: CAGAAATTCCGGGTAGATATGCC |
|  | R: GGTATGGGTTGGACATGGAGG |
| *LCN2* | F: GAAGTGTGACTACTGGATCAGGA |
|  | R: ACCACTCGGACGAGGTAACT |
| *ACSL4* | F: GCATTCCTCCAAGTAGACC |
|  | R: ATGAGCCAAAGGCAAGT |
| *PTGS2* | F: CTGGCGCTCAGCCATACAG |
|  | R: CGCACTTATACTGGTCAAATCCC |
| *FTH1* | F: AAGCTGCAGAACCAACGAGG |
|  | R: AGTCACACAAATGGGGGTCATT |
| *CD206* | F: TATGCCAGACACGATCCGACCC |
|  | R: AGTATGTCTCCGCTTCATGCCAT |
| *Arg1* | F: TGGACAGACTAGGAATTGGCA |
|  | R: CCAGTCCGTCAACATCAAAACT |
| *iNOS* | F: TCAGCTGTGCCTTCAACCC |
|  | R: CCGAGGCCAAACACAGCGTA |
| *β-actin* | Purchased from Biotechnology (Code No. B661102) |
